# Multi-Omics Data Analysis of Gene Expressions and Alterations, Cancer-Associated Fibroblast and Immune Infiltrations, Reveals the Onco-Immune Prognostic Relevance of STAT3/CDK2/4/6 in Human Malignancies

**DOI:** 10.3390/cancers13050954

**Published:** 2021-02-25

**Authors:** Bashir Lawal, Li-Ching Lin, Jih-Chin Lee, Jia-Hong Chen, Tanios S. Bekaii-Saab, Alexander T. H. Wu, Ching-Liang Ho

**Affiliations:** 1 PhD Program for Cancer Molecular Biology and Drug Discovery, College of Medical Science and Technology, Taipei Medical University, and Academia Sinica, Taipei 11031, Taiwan; d621108004@tmu.edu.tw; 2 Graduate Institute for Cancer Biology & Drug Discovery, College of Medical Science and Technology, Taipei Medical University, Taipei 11031, Taiwan; 3 Department of Radiation Oncology, Chi-Mei Foundation Medical Center, Tainan 71004, Taiwan; 8508a6@mail.chimei.org.tw; 4 Department of Otolaryngology-Head and Neck Surgery, Tri-Service General Hospital, National Defense Medical Center, 325 Cheng-Kung Road Section 2, Taipei 114, Taiwan; doc30450@mail.ndmctsgh.edu.tw; 5 Division of Hematology/Oncology, Department of Medicine, Tri-Service General Hospital, National Defense Medical Center, Taipei 114, Taiwan; wen17140@mail.ndmctsgh.edu.tw; 6 Division of Hematology and Medical Oncology, Mayo Clinic Arizona, Scottsdale, AZ 85054, USA; bekaii-saab.tanios@mayo.edu; 7 The PhD Program of Translational Medicine, College of Science and Technology, Taipei Medical University, Taipei 11031, Taiwan; 8 Clinical Research Center, Taipei Medical University Hospital, Taipei Medical University, Taipei 11031, Taiwan; 9 National Defense Medical Center, Graduate Institute of Medical Sciences, Taipei 114, Taiwan

**Keywords:** cyclin-dependent kinases, signal transducer and activator of transcription 3, genetic alterations, tumor immune infiltrations, cancer-associated fibroblast infiltration

## Abstract

**Simple Summary:**

Signal transducer and activator of transcription 3 (STAT3)/Cyclin-dependent kinases are multifunctional proteins that play instrumental roles in carcinogenesis. However, the genetic alterations of the STAT3/CDK2/4/6 signaling axis and its role in predicting immune infiltration and immunotherapeutic response remain unclear. Here, we used in silico analyses of multi-Omics data to map out the role of epigenetic and genetic alterations of STAT3/CDK2/4/6 in tumor immune infiltrations, immunotherapy response, and prognosis of cancer patients. Our study collectively suggested that STAT3/CDK2/4/6 are important onco-immune signatures that contribute to tumor immune invasion, poor prognoses, and immune therapy failure. Our finding may be clinically useful in designing therapeutic strategies, prognosis assessment, and follow-up management in patients receiving immunotherapy in multiple cancers.

**Abstract:**

Signal transducer and activator of transcription 3 (STAT3)/Cyclin-dependent kinases are multifunctional proteins that play an important implicative role in cancer initiations, progression, drug resistance, and metastasis, and has been extensively explored in cancer therapy. However, the genetic alterations of STAT3/CDK2/4/6 and its role in predicting immune infiltration and immunotherapeutic response are yet to be well exploited. In this study, we use in silico methods to analyze differential expression, prognostic value, genetic and epigenetic alterations, association with tumor-infiltrating immune cells, and cancer-associated fibroblast (CAF) infiltrations of STAT3/CDK2/4/6 in multiple cancer types. Our results revealed that the expression of STAT3/CDK2/4/6 was altered in various cancers and is associated with poor overall and disease-free survival of the cohorts. Moreover, genetic alterations in STAT3/CDK2/4/6 co-occurred with a number of other genetic alterations and are associated with poorer prognoses of the cohorts. The protein-protein interaction (PPI) network analysis suggests CDK2/4/6/STAT3 may directly interact with factors that promote tumorigenesis and immune response. We found that STAT3/CDK2/4/6 expressions were associated with infiltrations of CAF and the various immune cells in multiple cancers and it’s associated with poor response to immunotherapy. Collectively, our study suggested that STAT3/CDK2/4/6 are important onco-immune signatures that play central roles in tumor immune invasion, poor prognoses and, immune therapy response. Findings from the present study may therefore be clinically useful in prognosis assessment and follow-up management of immunotherapy.

## 1. Introduction

Early diagnosis of cancer increases the success of conventional therapies including surgery, chemo-, radio- and immunotherapy. However, cancers diagnosed at a later stage are more susceptible to treatment failure, drug resistance, metastasis, and poor prognosis [1]. Because most cancers are diagnosed at a later stage, the survival rate of patients is often low, less than 3 years in most cases. Therefore, cancer remains a public health concern and currently ranked the second leading cause of global mortality [2,3].

The role of genetic mutation as a biomarker in the diagnosis and prognosis of different cancers has been described in previous studies, providing insight into the developments of neoantigens for tumor immune invasion and cancer’s life-threatening physiognomies, such as incessant growth and metastasis [4,5]. Accumulating evidence indicates that the tumor microenvironment (TME) and immune cell infiltration play a key role in tumor progression and poor prognosis [6,7]. The accretion of various infiltrating immune cells such as regulatory T cells, natural killer cells, B-cells, and tumor-associated-macrophages, in the TME, has been found to be associated with tumor progression. These infiltrating immune cells, do not inhibit the growth of cancer cells but play a crucial role in mediating tumor immune escape [8,9]. Immune checkpoints such as cytotoxic T lymphocyte-associated antigen 4 (CTLA4) and programmed cell death ligand-1 (PD-L1) have been shown to impede anti-tumor immunity, leading to the invasion of host immune attack [10,11]. Already, several biomarkers have been incorporated into clinical practice, the outcome of cancer immunotherapy is still disappointing [8]. Therefore, finding novel potential targets for cancer immunotherapy and biomarkers for effective screening in the earlier stages can be a powerful tool to improve long-term survival [12,13].

Cyclin-dependent kinases (CDKs) are serine/threonine enzymes of cell cycle checkpoints whose catalytic activities are controlled by occasional complexation of its catalytic unit with its regulatory unit, cyclins [14]. CDKs are multi-functional proteins whose role includes metabolism, epigenetic regulations, spermatogenesis cell cycle transition, and stem cell self-renewal [15,16]. Clinical studies also indicated that CDKs regulate pro-inflammatory response by mediating pro-inflammatory transcription factors such as the signal transducer and activator of transcription 3 (STAT3) and nuclear factor kappa B [17]. STAT3 is also a multifunctional transcriptional factor with an important implicative role in cancer progression and drug resistance [18,19] and has been extensively explored in cancer therapy [20,21]. However, its role in predicting immune infiltration and immunotherapeutic response is yet to be well exploited. Among the CDKs, CDK1/2/4/6 are particularly important in regulating cell cycle transition. They regulate cell cycle transition via phosphorylation and inactivation of various regulatory proteins such as cell cycle inhibitors Whi5 [22] and retinoblastoma (Rb), a cell cycle inhibitor and tumor suppressor protein [23]. However, epigenetic factors and genetic factors including the loss of cyclin D-CDK4/6 negative regulators, overexpression of cyclin D, amplification and/or mutation of CDK4/6, compromises the regulatory integrity of the CDKs leading to hyper complexation of the catalytic and regulatory unit and consequently un-control cell cycle progression, cancer initiation and developments [23,24,25]. Aberrant CDKs expressions, therefore, constitute an important event in cancer development, progression, and aggressiveness. Altogether, identifying the association between CDK2/4/6/STAT3 and infiltration of various immune cells will help in developing an important biomarker for early stratification of patient immune status and response to immunotherapy.

The use of bioinformatics for the identification of important cancer biomarkers is increasingly becoming a reliable and profitable method [26,27], owing to the availability of multi-omics clinical data including differentially expressed genes, mutation profile, therapeutic response, and survival profile of cancer patients in public databases providing a reliable guideline for the development of appropriate therapeutic intervention [12]. In addition, network analysis of multi-omics data has also helped our understanding of the epigenetic mechanism of cancer development and facilitated the discovery of epigenetic-based prognostic biomarkers and therapies [28,29,30,31,32]. In this study, we identified STAT3/CDK2/4/6 as an oncogenic prognosticator of cancer-associated fibroblasts and tumor immune infiltrations. We also demonstrated that the STAT3/CDK2/4/6 signature is associated with immune therapy response and poor prognosis of multiple cancer cohorts. Genetic alteration of STAT3/CDK2/4/6 co-occurred with other gene alteration and are associated with poorer prognosis of the cohorts. Our finding may be clinically useful in designing appropriate therapeutic strategies, prognosis assessment, and follow-up management of immunotherapy in multiple cancers.

## 2. Materials and Methods

### 2.1. Differential Expression Analysis of STAT3/CDK2/D/6 Signatures in a Panel of Human Cancers

We used the Tumor IMmune Estimation Resource (TIMER2.0) algorithm (http://timer.cistrome.org/, accessed on 13 December 2020) to compare the STAT3, CDK2, CDK4, and CDK6 expression levels between tumor tissues and matched normal tissues in The Cancer Genome Atlas (TCGA) database. Furthermore, immunohistochemical data from the Human Protein Atlas (HPA) database (www.proteinatlas.org, accessed on 13 December 2020) was used to analyze CDK2, CDK 4, CDK6, and STAT3 expressions in tumor samples from cancer patients.

### 2.2. Survival Analysis of STAT3/CDK2/D/6 Signature in a Panel of Human Cancers

To analyzed the prognostic value of STAT3/CDK2/D/6 signature, we collected the RNA expression profile of STAT3/CDK2/4/6 signature from the 9736 tumor samples across 33 TCGA and GTEx datasets using the Gene Expression Profiling Interactive Analysis (GEPIA) database (http://gepia.cancer-pku.cn/, accessed on 15 December 2020) [33] and then set the median expression as the expression threshold to split the patient samples into high-STAT3/ CDK2/4/6 high-expression and low-expression groups, and used the Kaplan-Meier survival plot to assess the overall survival (OS) and disease-free survival (DFS) with the hazard ratio (HR), a 95% confidence interval (CI), and a log-rank test *p*-value.

### 2.3. Protein-Protein Interaction and Functional Enrichment Analysis

The protein-protein interaction (PPI) network and the functional enrichment analysis including Kyoto Encyclopedia of Genes and Genomes (KEGG) pathways and Gene Ontology (GO); biological process and clinical pathology enriched in STAT3/CDK2/4/6 PPI network were analyzed using the Search Tool for Retrieval of Interacting Genes (STRING, version 10.5, (https://www.string-db.org/, accessed on 24 December 2020) with the adjusted threshold confidence set at 0.900 [34] and Enrich (https://maayanlab.cloud/Enrichr/enrich#, accessed on 24 December 2020) [35,36].

### 2.4. Analysis of STAT3/CDK2/4/6 Genetic Alterations and Its Prognostic Relevance in Multiple Cancers

We explore the cancer genomic data set through the cBioPortal tool (http://www.cbioportal.org/, accessed on 26 December 2020) to analyze the genomic alterations, survival analysis, gene alteration co-occurrence, and perform group comparisons of STAT3/CDK2/4/6 in 10,953 cancer patient (10,967 samples) from different cancer types [37,38]. While we used the Tumor Immune Dysfunction and Exclusion (TIDE) (http://tide.dfci.harvard.edu, accessed on 15 February 2021) tools [39] to analyze the copy number alterations (CAN) data together with both survival durations and tumor gene expression profiles of cancer cohorts from 36 cancer types consisting of 30 TCGA cancers datasets [40] and six METABARIC breast cancer subtypes (luminal A, luminal B, Her2 positive, basal, and triple-negative) datasets [41] through the TIDE server. All the analysis was considered significant at *p* < 0.05.

### 2.5. Analysis of STAT3/CDK2/4/6 Association with Infiltrations of Cancer-Associated Fibroblast and Various Immune Cells

We also used the TIMER algorithm to comprehensively analyze correlations between STAT3/CDK2/4/6 expressions and six tumor-infiltrating immune cell subsets (B cells, CD4 T cells, CD8 T cells, macrophages, neutrophils, and dendritic cells) in multiple cancers from the TCGA database [42]. We used the purity adjustment and partial Spearman’s correlation to analyzed the STAT3/CDK2/4/6 expression correlations with cancer-associated fibroblast (CAF) across 40 TCGA cancer types using the TIMER server. To evaluate the prognostic relevance of these associations, we classified all cohorts into 4 groups; ^low^CAF + ^low^STAT3/CDK2/4/6, ^low^CAF + ^high^STAT3/CDK2/4/6, ^high^CAF + ^low^STAT3/ CDK2/4/6, and ^high^CAF + ^high^STAT3/CDK2/4/6 and used the Kaplan-Meier survival plot to analyzed the cumulative survival of the cohorts.

### 2.6. Analysis of STAT3/CDK2/4/6 Association with Dysfunctional T-Cells and Clinical Outcome of Immunotherapy

To determine the association between STAT3/CDK2/4/6 DNA methylation and dysfunctional T-cell phenotype, and survival of cancer patients, we analyzed the promoter DNA methylation data of STAT3/CDK2/4/6 together with the survival durations and tumor gene expression profiles of 30 TCGA cancer types using the TIDE server. All the analysis was considered significant at *p* < 0.05. In order to obtain the relationship between the STAT3/CDK2/D/6 signature and immunotherapy response, we used the Tumor Immune Dysfunction and Exclusion (TIDE) (http://tide.dfci.harvard.edu, accessed on 15 February 2021) tools [39] to analyze the correlation between the expression of these signatures and the therapy outcome in clinical studies of immune checkpoint blockade in patients with brain cancer and melanoma. We obtained the transcriptomic and clinical data with the response to anti-PD1 ICB [43] or anti-CTL4A [44] treatments in melanoma patients and anti-PD1 ICB treatment in brain cancer [45] patients. We divided these patients into high-STAT3/CDK2/D/6 expression and low- STAT3/CDK2/D/6 expression groups according to the median expression of these genes, respectively, and assessed the OS of patients by using a Kaplan-Meier survival plot.

### 2.7. Statistical Analysis

Spearman’s rank correlation was used to assess the correlations of CDK2/CDK4/CDK6/STAT3 expressions with cancer-associated fibroblast and tumor immune infiltrations. The statistical significance of differentially expressed genes was evaluated using the Wilcoxon test. * *p* < 0.05; ** *p* < 0.01; *** *p* < 0.001. The Kaplan-Meier curve was employed to present the patients’ survival from different cancer cohorts. Gene alteration co-occurrence was calculated based on the cbioportal server instructions. The adjusted value < 0.05 was considered statistically significant.

## 3. Results

### 3.1. Overexpression of STAT3/CDK2/4/6 Signaling Networks Is Associated with Poor Prognoses of Multiple Cancers

**Figure 1:**
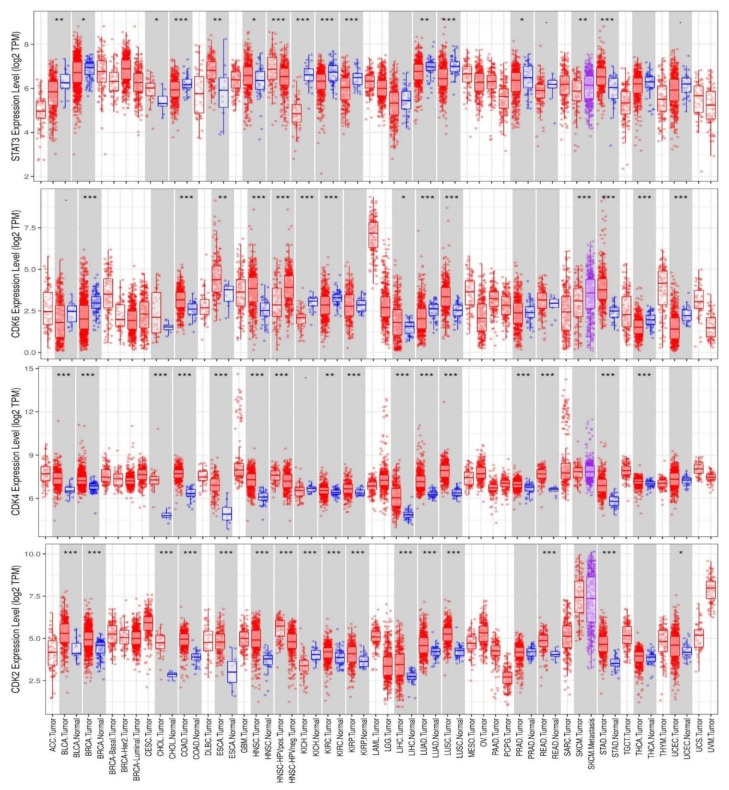
STAT3/CDK2/4/6 are overexpressed in multiple cancers. Box plots showing differential gene expression levels (Log2 TPM) of STAT3/CDK2/4/6 between tumor and adjacent normal tissues across TCGA database. Blue labels indicate normal tissues, and red labels indicate tumor samples. The statistical significance of differentially expressed genes was evaluated using the Wilcoxon test. * *p* < 0.05; ** *p* < 0.01; *** *p* < 0.001.

**Figure 2:**
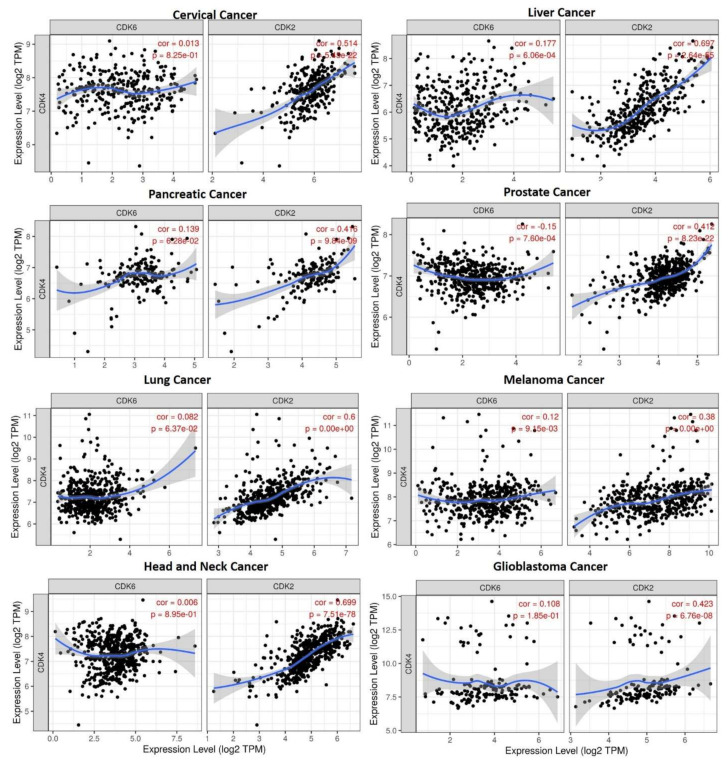
Expression scatterplots of CDK4 correlations with CDK2 and CDK6 in multiple cancer types. CDK4 expression was positively correlated with expressions of CDK2 and CDK6 in liver cancer, lung cancer, prostate cancer, pancreatic cancer, melanoma, head and neck cancer, glioblastomas, breast cancer, and cervical cancer cohorts (*r* = 0.06~0.69). CDK4 was negatively correlated with CDK6 expression in prostate cancer (*r* = −0.5). The strength of correlations between the genes is reflected by the purity-adjusted partial spearman’s rho value and estimated statistical significance, where a value of *r* = 1 means a perfect positive correlation and a value of *r* = −1 means a perfect negative correlation.

**Figure 3:**
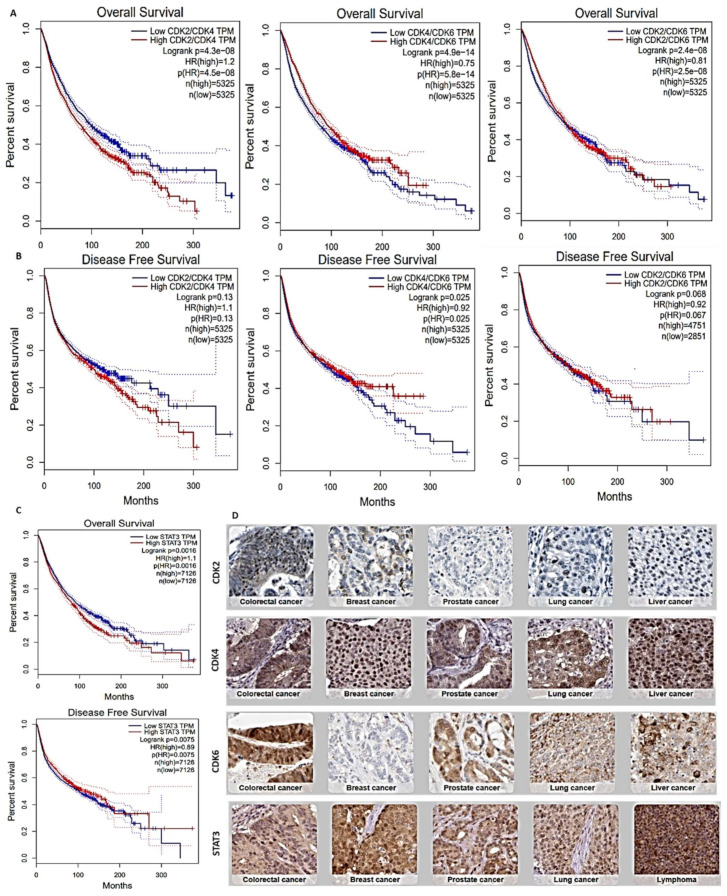
STAT3/CDK2/4/6 over expressions are associated with poor prognoses of cancer patients. Kaplan-Meier curve of (**A**) overall survival (upper panel) and (**B**) disease-free survival (lower panel) of cancer patients with low and high CDK2/4/6 and (**C**) STAT3 expressions across TCGA and GTEx datasets. Higher RNA expression profiles of STAT3/CDK2/4/6 correlated with low overall survival and disease-free survival of cancer patients. (**D**) Representative immunohistochemistry of STAT3/CDK2/4/6 staining across the Human Protein Atlas (HPA) database shows high intensities of CDK2 (antibody: CAB013115), CDK4 (antibody: CAB013116), CDK6, (antibody: HPA002637) and STAT3 (antibody: HPA001671) expressions in clinical samples.

Taking advantage of clinical data in The Cancer Genome Atlas (TCGA), we employed the DiffExp module of the TIMER server to identify CDK2/4/6 and STAT3 expressions in tumors and healthy cohorts across TCGA datasets. We found that CDK2, CDK4, CDK6, and STAT3 expressions were higher in tumor cohorts compared to normal cohorts (Figure 1). In particular, glioblastoma, breast cancer, colon cancer, melanoma, lung adenocarcinoma, head and neck cancer, pancreatic cancer, liver cancer, and prostate cancer cohorts showed the most significantly (*p* < 0.001) elevated CDK2, CDK4, CDK6, and STAT3 expressions. Correlation analyses also indicated that CDK4 expression was positively correlated with expressions of CDK2 and CDK6 in liver cancer, lung cancer, prostate cancer, pancreatic cancer, melanoma, head and neck cancer, glioblastoma, breast cancer, and cervical cancer cohorts (*r* = 0.06~0.69). The only exception was the negative correlations of CDK4 and CDK6 (*r* = −0.5) with prostate cancer (Figure 2). We carried out a survival analysis of RNA expression data from 9736 tumor cohorts of TCGA and GTEx datasets on GEPIA (http://gepia.cancer-pku.cn/index.html, accessed on 15 December 2020). Interestingly, we found that higher RNA expression profiles of CDK2/CDK4/CDK6 (Figure 3A) and STAT3 (Figure 3B) predicted significantly lower overall survival (OS) and disease-free survival (DFS). Furthermore, our exploration of the Human Protein Atlas (HPA) database for immunohistochemical (IHC) data of CDK2, CDK4, CDK6, and STAT3 expressions in tumor cohorts (Figure 3C, Table 1) revealed high staining intensities of CDK2 (antibody; CAB013115) in colorectal cancer (100%), head and neck cancer (100%), lung cancer (60.00%), glioblastoma (63.63%), prostate cancer, and pancreatic cancer datasets (50.00%); of CDK4 (antibody; CAB013116) in lung cancer (100%), colorectal cancer (100%), head and neck cancer (100%), breast cancer and glioblastoma (81.81%), prostate cancer (90.90%), pancreatic cancer (63.63%), and liver cancer datasets (66.66%); and of CDK6 (antibody; HPA002637) in lung cancer (36.36%), colorectal cancer (90.0%), head and neck cancer (100%), breast cancer (25.0%), glioblastoma (91.66%), prostate cancer (20.0%), pancreatic cancer (63.63%), and liver cancer datasets (75.00%); while STAT3 (antibody; HPA001671) was positively stained in more than 50% of all tumor samples in the HPA (Figure 3C, Table 1).

### 3.2. STAT3/CDK2/4/6 Are Enriched in Cancer and Immune Associated Signaling Networks

**Figure 4:**
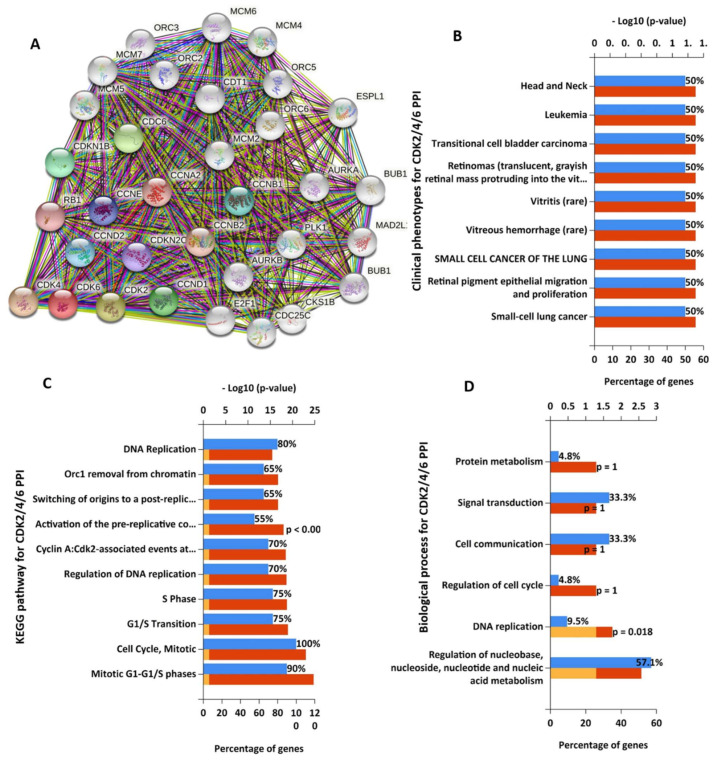
CDK2/4/6 clustering network revealed multiple interactions with oncogenic proteins. (**A**) The clustering network of CDK2/4/6 interactions generated a total of 33 nodes and 429 edges with an average local clustering coefficient of 0.877 and protein-protein interaction (PPI) enrichment *p*-value of <10^−16^. Enriched (**B**) clinical phenotypes, (**C**) KEGG pathways, and (**D**) biological processes for CDK2/4/6 clustering networks.

**Figure 5:**
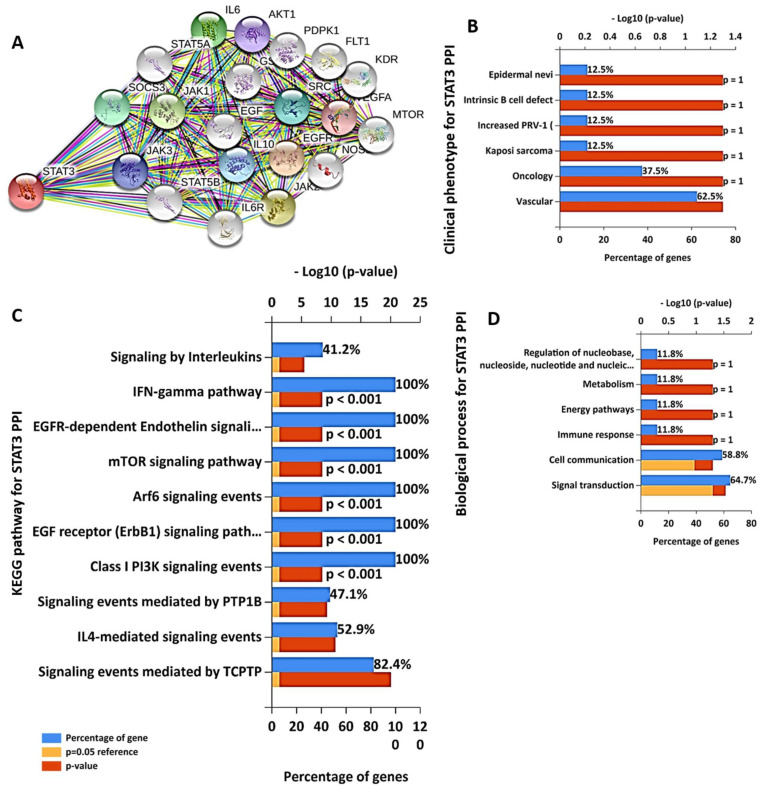
STAT3 clustering network revealed multiple interactions with oncogenic proteins. (**A**) Clustering network of STAT3-associated protein interactions. STAT3-associated protein interactions generated a total of 21 nodes and 161 edges with an average local clustering coefficient of 0.873 and protein-protein interaction (PPI) enrichment *p*-value of <10^−16^. Interactions are depicted at the highest confidence limit (0.900). Enriched (**B**) clinical phenotypes, (**C**) KEGG pathways, and (**D**) biological processes for STAT3 clustering networks.

The CDK2/4/6 clustering network of protein-protein interactions (PPIs) generated a total of 33 nodes and 429 edges with an average local clustering coefficient of 0.877 and a PPI enrichment *p*-value of <10^−16^ (Figure 4A). However, CDK2, 4, and 6 directly interacted with 20, 17 and 15 proteins with interactive scores ranging 0.453~0.99 (Supplementary Table S1). As shown in Figure 4B, the top most enriched clinical phenotypes in CDK2/4/6 PPI networks are head and neck cancer, leukemia, bladder carcinoma, vitrities, and small cell lung cancer. The most enriched Kyoto Encyclopedia of Genes and Genomes (KEGG) pathways included cell cycle, DNA replication, and activation pre-replicative pathways (Figure 4C). while protein metabolism, signal transductions, cell communications regulations of the cell cycle, and DNA replication are the most enriched biological process (Figure 4D). The STAT3 clustering network generated a total of 21 nodes and 161 edges with an average local clustering coefficient of 0.873 and a PPI enrichment *p*-value of <10^−16^ (Figure 5A). The most interactive proteins with STAT3 were AKT, epidermal growth factor receptor (EGFR), interleukin (IL)-6, IL-10, and Janus kinase 1/2/3 (JAK1/2/3) (0.994~0.995) (Supplementary Table S2). The most enriched clinical phenotypes in STAT3 clustering networks were, Kaposi’s sarcoma-associated herpesvirus infection, pathways in oncology, and the B-cell defect (Figure 5B), while the top enriched KEGG were IL-4, PI3K, TCPTP, EGFR, IFN-gamma, and mTOR-mediated signaling pathway, (Figure 5C), while signal transductions, cell communications, and immune response were enriched biological process (Figure 5D).

### 3.3. STAT3/CDK2/4/6 Expressions Are Associated with Tumor Immune Infiltrations

Tissue immune infiltration is highly involved in immune reactions [46]; therefore, considering the interactive networks and prognostic values of CDK2/4/6 and STAT3, we reasoned that CDK2/4/6 and STAT3 would more likely be highly expressed in immune cells and be correlated with tumor immune cell infiltration. Therefore, we explored associations of CDK2/4/6/STAT3 expressions with infiltrating immune cells (CD4+ T cells, B cells, CD8+ T cells, neutrophils, dendritic cells, and macrophages) in multiple cancers. Our results revealed that STAT3 expression was positively correlated with infiltration of macrophages, dendritic cells, CD4+ T cells, CD8+ T cells, neutrophils, and B cells (all *r* > 0.06, *p* < 0.005) in all cancer types analyzed, with the exception of glioblastoma multiforme (GBM). STAT3 expression was negatively correlated with B-cell and CD8+ T cell infiltration in GBM. Furthermore, all tumor analyses showed negative STAT3 correlations with tumor purity (Supplementary Figure S1, Table S2). CDK2/4/6 expressions were positively correlated with levels of different infiltrating immune cell types in multiple cancers (Supplementary Figures S2–S4, Table S2). Specifically, CDK2 expression was positively correlated with macrophages (all *r* > 0.03284, *p* < 0.002), dendritic cells (all *r* ≥ 0.13, *p* < 0.0003), CD4+ T cells (all *r* > 0.07, *p* < 0.002), CD8+ T cells (all *r* > 0.09, *p* < 0.004), neutrophils (all *r* > 0.21, *p* < 0.001), and B cells (all *r* > 0.11, *p* < 0.000) in lung adenocarcinomas, liver hepatocellular carcinoma, head and neck cancers, and breast invasive carcinoma (Supplementary Figure S2, Table 2). CDK4 expression was positively correlated with macrophages (all *r* > 0.19, *p* < 2.47 × 10^−5^), CD4+ T cells (all *r* > 0.04, *p* < 0.019), CD8+ T cells (all *r* > 0.03, *p* < 0.03), neutrophils (all *r* > 0.09, *p* < 0.0031), dendritic cells (all *r* ≥ 0.11, *p* < 0.0003), and B cells (all *r* > 0.08, *p* < 0.006) (Supplementary Figure S3, Table 2). CDK6 expression was positively correlated with macrophages only in breast cancer (BRCA), liver hepatocellular carcinoma (LIHC), and lung adenocarcinomas (LUADs) (Supplementary Figure S4, Table 1). However, CDK2/CDK4 and CDK6 expressions were negatively correlated with immune infiltration in glioblastomas and skin cutaneous melanomas. Furthermore, CDK6 was negatively correlated with tumor purity in BRCA (*r* = −0.31833, *p* = 7.21 × 10^−25^), head and neck squamous cell carcinomas (HNSCs) (*r* = −0.06379, *p* = 0.157308), LIHC (*r* = −0.11342, *p* = 0.034946), and LUADs (*r* = −0.16182, *p* = 0.000304). However, CDK2 and CDK4 were positively correlated with tumor purity in skin cutaneous melanomas, LUADs, LIHCs, head and neck cancers, glioblastomas, and breast invasive carcinoma. Altogether, the above results indicate that STAT3/CDK2/4/6 genes are differentially expressed within the TME and in tumor cells. They also exhibited varied and tumor-dependent correlations with immune infiltration and thus may be involved in the immune response within the TME.

Having established the negative correlation of tumor immune infiltration with CDK2/4/6 expression in GBM and melanoma, we wonder if there will be an association between these genes alterations and immune infiltration, to this ends we analyzed the correlation between different somatic copy number alterations of CDK2/4/6/STAT3 and immune cell infiltration in glioblastoma and melanoma. The results showed that the arm−level gain and high amplification of CDK2 in GBM was negatively (*p* < 0.05) associated with B-cell, macrophages, CD4+ T cell dendritic cell infiltration (Supplementary Figure S5), while CDK4 SCNAs showed no or weak relationship with infiltration of the above six immune cell types. Conversely, Arm level gain of CDK6 shows a positive (*p* < 0.05) correlation with increase CD4+ T cell, CD4+ T cell, macrophages, and dendritic cell infiltration, while arm level deletion shows a strong negative correlation (*p* < 0.001) with B-cell infiltrations in GBM patient. Similarly, arms level gain of STAT3 shows was negatively associated with CD4+ T cell and dendritic cell infiltration. We also found a negative association of CDK2/4/6/STAT3 SCNA with the various immune infiltration in melanoma (Supplementary Figure S5).

### 3.4. STAT3/CDK2/4/6 Are Associated with Cancer-Associated Fibroblast (CAF) Infiltration

**Figure 6:**
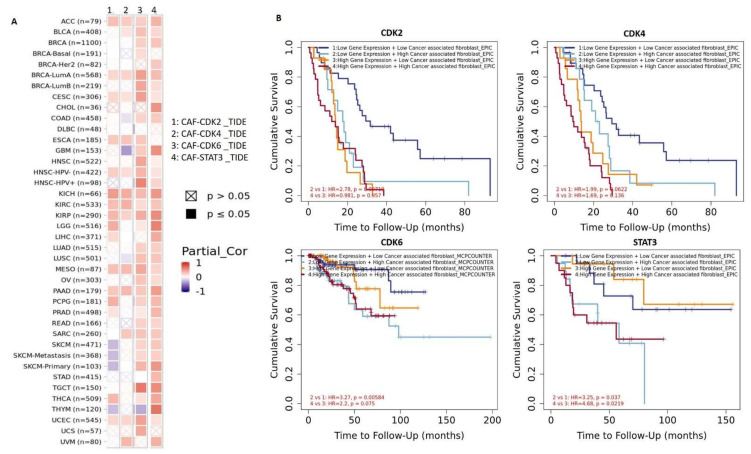
STAT3/CDK2/4/6 expressions were associated with cancer-associated fibroblast (CAF) infiltration (**A**). Heat map showing correlations of STAT3/CDK2/4/6 expressions and CAF infiltration in multiple cancer types. Out of the 40 cancer types presented in the heat map, patients of 36 cancer types exhibited correlations of CAF and STAT3 expressions, while 34 exhibited correlations of CAF and CDK6 expressions. Patients of 14 cancer types exhibited correlations of CDK2 and CDK4 with CAFs (**B**). Kaplan-Meier curve of cumulative survival of cancer cohorts with CAF-STAT3/CDK2/4/6 associations. All cohorts were grouped into 4; ^low^CAF + ^low^STAT3/CDK2/4/6, ^low^CAF + ^high^STAT3/CDK2/4/6, ^high^CAF + ^low^STAT3/CDK2/4/6, and ^high^CAF + ^high^STAT3/CDK2/4/6.

We analyzed CAF correlations with expression profiles of STAT3/CDK2/4/6 and found that out of the 40 TCGA cancer types analyzed via the TIMER server, CAF and STAT3 expressions were correlated in cohorts of 36 cancers types, while CAF and CDK6 expressions were correlated in 34 cancer types. The strongest CAF-STAT3 association (Partial cor = 1, *p* < 0.05) was observed in cholangiocarcinoma (CHOL), GBM, kidney chromophobe (KICH), kidney renal papillary cell carcinoma (KIRP), low-grade glioma (LGG), pancreatic adenocarcinoma (PAAD), thymoma (THYM), testicular germ cell tumor (TGCT) and primary SKCM. However, no significant CAF-STAT3 association (*p* > 0.05) was observed in cohorts of diffuse large B-cell lymphoma (DLBC), esophageal carcinoma (ESCA), uterine corpus endometrial carcinoma (UCS), and basal breast invasive carcinoma (BRCA) (Figure 6A). CAF-CDK6 association was highly correlated (Partial cor = 1, *p* < 0.05) in cohorts of BRCA-Luma A, BRCA-Luma B, CESC, HNSC-HPV+, and TGCT. However, expressions of CDK2 and that of CDK4 correlated with CAF infiltrations in cohorts of 14 cancer types. To evaluate the prognostic relevance of these associations, we classified all cohorts into four groups; ^low^CAF + ^low^STAT3/CDK2/4/6, ^low^CAF + ^high^STAT3/CDK2/4/6, ^high^CAF + ^low^STAT3/CDK2/4/6, and ^high^CAF + ^high^STAT3/ CDK2/4/6. Interestingly, we found that the cohort in ^high^CAF + ^high^STAT3/CDK2/4/6 group exhibited low cumulative survival than cohorts of other groups (Figure 6B).

### 3.5. Genetic Alterations of STAT3/CDK2/4/6 Are Associated with Poor Prognosis

**Figure 7:**
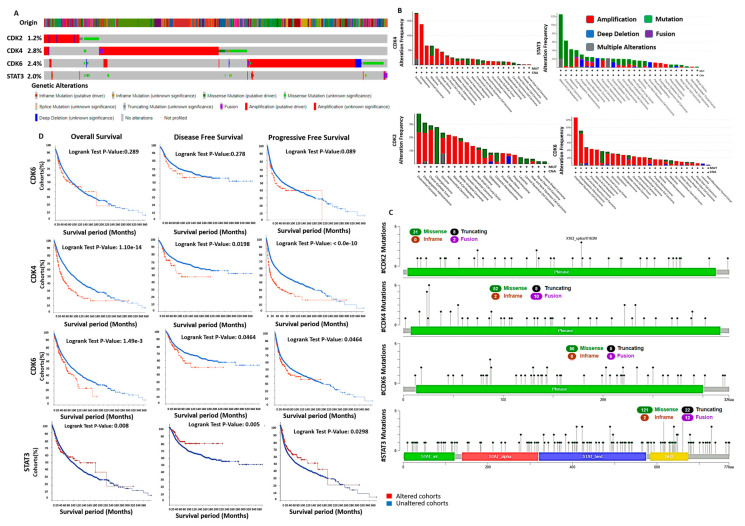
Genetic alterations of STAT3/CDK2/4/6 are associated with poor prognosis (**A**) Prevalence and distribution of STAT3/CDK2/4/6 genetic alterations in cancer patient across the cBioPortal for Cancer Genomics dataset. (**B**) STAT3/CDK2/4/6 alteration frequency across cancer types. The types of alterations are color-coded as shown in the legend above. (**C**) Lollipop plot STAT3/CDK2/4/6 mutation types in cancer patients across the cBioPortal for Cancer Genomics dataset. Mutations are color-coded as missense, truncating, and inframe mutations. STAT_INT: STAT protein-protein interaction domain, STAT_alpha: STAT all-alpha domain, STAT_bind: STAT DNA binding domain, SH2: SH2 domain. (**D**) Kaplan Meier curve of the overall survival, disease free survival and progressive free survival in CDK2, CDK4, CDK6 altered and non-altered cancer cohorts.

Among, 10953 patients/10967 samples of all type of human cancers publicly available in the online cancer genomic database cBioPortal, genetic alterations of CDK2/4/6 and STAT3 occurs in 825 (8%) patients, comprising 127 (1.2%) CDK2, 307 (2.8%) CDK4, 266 (2.4%) CDK6, and 220 (2%) STAT3 and (Figure 7A). The CDK2 alterations (127; 1.2%) occur in 22 cancer types, mostly in endometrial carcinoma (4.1%), esophagogastric carcinoma (3.11%), and ovarian epithelial tumor (2.91%). The most common alterations in CDK2 is amplification (85 cases, 66.92%), mutation (35 cases, 27.55%), while deep deletion (four cases, 3.14%), and multiple alterations (three cases, 2.36%) are the least occurred (Figure 7B). The CDK4 alterations occur in 23 cancer types, mostly in sarcoma (17.65%), glioblastoma (13.85), and adrenocortical carcinoma (6.59%). Amplification (246 cases, 79.15%), and mutation (55 cases, 17.91%), are the most common CDK alterations while multiple alterations (eight cases, 2.60%) and deep deletion (one case, 0.32%) are least occurred. CDK6 alterations occur in 26 cancer types, mostly in esophageal squamous cell carcinoma (95, 12.63%), esophagogastric adenocarcinoma (514 cases, 9.14%), and head and neck squamous cell carcinoma (523 cases, 4.78%). The most common alterations in CDK6 is amplification (199 cases, 74.81%), mutation (46 cases, 17.29%), deep deletion (13 cases, 4.88%) while multiple alterations (seven cases, 2.63%) and fusion (one case, 0.37%) occurred the least. The STAT3 alterations occur in 27 cancer types, comprising of mutation (136 cases, 61.81%), amplification (48 cases, 21.81%), deep deletion (25 cases, 11.36%), fusion (seven cases, 3.18%), and multiple alterations (four cases, 1.81%) occurred the least (Figure 7B). Specific mutation profiling indicated that out of the total CDK2 mutation in the database, 75.60% were missense, 19.51%, were truncating while 4.87% cases were fusion mutations (Figure 7C). For CDK4 mutation, 52 (75.36%) were missense, 10 (14.49%) fusion, five (7.24%) truncating while two (2.89) cases were inframe mutations). Of the total CDK6 mutation in the database, 81.96%, 8.19%, and 9.83% were missense, truncating, and fusion, respectively (Figure 7C).

We analyzed the prognostic relevance of CDK2, CDK4, and CDK6 genetic alterations and found that CDK4 and CDK6 alterations are associated with low overall survival, disease-free survival, and progression-free survival of cancer cohorts (*p* < 0.05). However, genetic alteration in CDK2 was not associated (*p* > 0.05) with low overall survival, disease-free survival, and progression-free survival of the cohorts (Figure 7D).

### 3.6. Enrichment of Genes Alteration Co-Occurrence in Cancer Cohorts with STAT3/CDK2/4/6 Alterations

**Figure 8:**
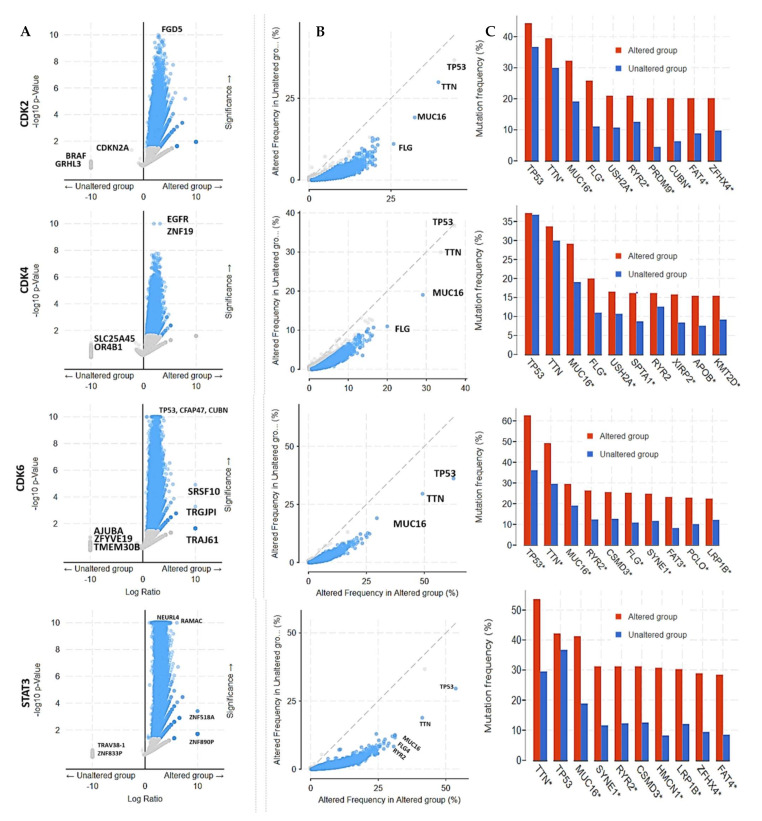
Enrichment frequencies of gene alteration co-occurrence in cancer cohorts with altered and those with non-altered STAT3/CDK2/4/6. (**A**): Scatter plot of the significantly enriched co-occurred gene alterations in STAT3/CDK2/4/6 altered, and non-altered cancer cohorts. (**B**) Scatter plot of the frequencies of all co-occurred gene alterations in STAT3/CDK2/4/6 altered, and non-altered cancer cohorts. (**C**) Bar plot showing the top 10 most frequently mutated genes in STAT3/CDK2/4/6 altered, and non-altered cancer cohorts.

We also analyzed the frequency of gene alteration co-occurrence with CDK2, CDK4, CDK6, and STAT3 genetic alteration (Figure 8A,B), and found co-occurrence of genetic alterations in a total of 19434 genes, enriched in CDK2/4/6 and STAT3 altered and non-altered cohorts. The different frequencies of alterations in the co-occurred genes are shown in Figure 8B. However, only, 12676, 9265, 14130, and 17416 altered genes were significantly enriched in CDK2, CDK4, CDK6, and STAT3 altered cohorts respectively, while no gene alteration was significantly (all *p* > 0.05) enriched in CDK2/4/6/STAT3 unaltered cohorts (Figure 8A). The top 10 altered genes with significant enrichment in CDK2 (all *p*-value < 1 × 10^−9^), CDK4 (all *p*-value < 1 × 10^−7^), CDK6 (all *p*-value < 1 × 10^−11^) and STAT3 (all *p*-value < 1 × 10^−19^) altered cohorts are presented in Table 3. However, TP53, TTN, MUC16, and FLG were the most frequently mutated genes in all CDK2, CDK4, and CDK6 altered and non-altered cohorts, while TTN, TP53, MUC16, SYNE1, RYR2, CSMD3, HMCN1, LRP1B, ZFHXA, and FAT4 are the most frequently mutated genes in both STAT3 altered and non-altered cohorts (Figure 8C).

### 3.7. DNA Methylation and Copy Number Alterations of STAT3/CDK2/4/6 Are Associated with Dysfunctional T-Cell Phenotypes and Are of Prognostic Relevance in Multiple Cancers

**Figure 9:**
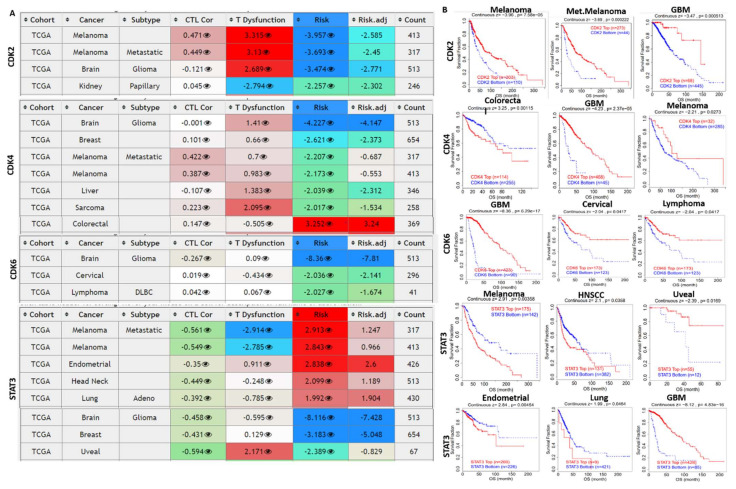
DNA methylation of STAT3/CDK2/4/6 are associated with dysfunctional T-cell phenotypes and are of prognostic relevance in multiple cancers. (**A**) Graphical data representation of the effect of STAT3/CDK2/4/6 DNA methylation on cytotoxic lymphocyte infiltrations, dysfunctional T-cell phenotypes and risk factor in different TCGA cancer types and subtypes. The deeper the red color and higher positive value indicate poor prognosis with respect to hyper-methylation of STAT3/CDK2/4/6 while the blue to green color indicate poor prognosis with respect to hypo-methylation of STAT3/CDK2/4/6. (**B**) Kaplan Meier curve of the overall survival difference between cancer cohorts with hyper-DNA methylation and hypo-DNA methylation of STAT3/CDK2/4/6.

Analysis of the promoter DNA methylation indicated that among 30 TCGA cancer type hypo-methylation of CDK2 are significantly associated with T cell dysfunctional phenotype high death risk and shorter survival durations in melanoma, kidney, and brain cancer only (Figure 9A). Similarly, hypo-methylation of CDK2 is associated with T cell dysfunctional phenotype and worse prognosis of the brain, melanoma, metastatic melanoma, liver, and sarcoma patient while in colorectal cancer patients, it shows a negative association with dysfunctional T cells and predicts a good prognosis of the cohorts (Figure 9A). Hypo-methylation of CDK6 is associated with T cell dysfunctional phenotype high death risk and low survival duration in lymphoma, cervical, and brain cancer patients (Figure 9A,B). Hyper methylation of STAT3, on the other hand, predicted high death risk and poor survival of melanoma, metastatic melanoma, endometrial, head and neck cancer, and lung cancer patients while predicting low death risk and longer survival duration in the brain, breast, and uveal cancers (Figure 9B). Copy number alteration of CDK2 is associated with dysfunctional T-cell phenotype, high death risk, and shorter survival of lymphoma, leukemia, and breast cancer patients while CDK4 predicted a worse prognosis of the brain, lymphoma, and breast cancer patients. CNA of CDK6 on the other hand predicted a worse prognosis in the brain and a good prognosis of breast cancer cohorts. STAT3 CNA predicted dysfunctional T cell phenotype and worse prognosis in stomach and lymphoma cancers while predicting a good prognosis of endometrial and brain cancer (Supplementary Figure S6).

### 3.8. STAT3/CDK2/4/6 Overexpression Predicts Poor Clinical Benefit to Immune Checkpoint Blockade Therapy

**Figure 10:**
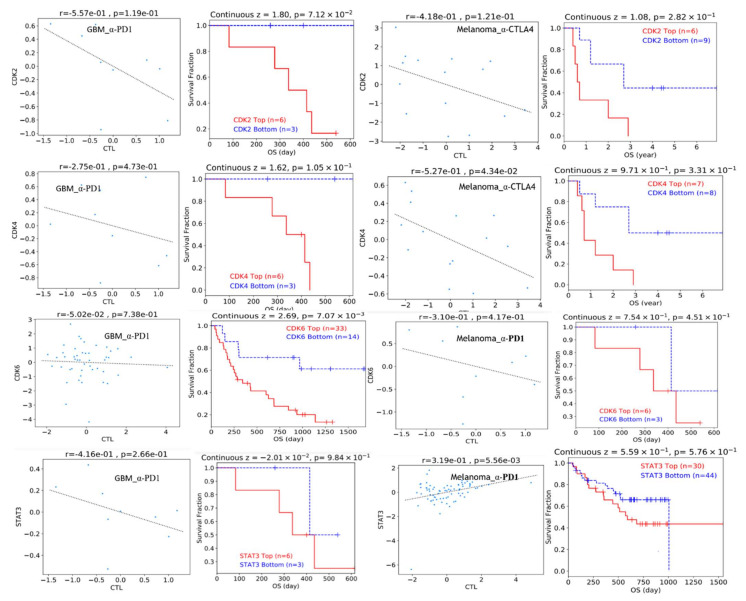
Effect of STAT3/CDK2/4/6 expression on therapy outcome in clinical studies of immune checkpoint blockade. Scatter plot of Spearman’s rank correlation and Kaplan Meier curve of the overall survival of cancer cohorts with up-expressed and down expressed STAT3/CDK2/4/6 status treated with anti-PD1 or anti-CTLA4 therapy.

We used the transcriptomic and clinical data from melanoma and glioblastoma patients of anti-PD1 or anti-CTLA4 therapy to predict the immunotherapy response of cohorts with different STAT3/CDK2/4/6 expression. In agreement with our earlier observation, we found that the expression of CDK2/4/6 shows a negative correlation with cytotoxic lymphocyte infiltration (CTL) in both GBM and melanoma, while STAT3 shows a negative correlation with CTL infiltrations in GBM but a positive correlation in melanoma. In addition, patients with higher expression of STAT3/CDK2/4/6 signature exhibited a poor response to anti-PD1 or anti-CTLA4 therapy and exhibited shorter survival than patients with low expression profiles (Figure 10).

## 4. Discussion

Despite advances in treatment modalities, cancer survival ratios are still disappointing; thus, improving survival rates in cancer patients remains a global research focus [47,48,49,50,51]. Identifying prognostic markers for immune response and poor survival rates in cancer is an important preceding aspect for developing adequate therapeutic interventions. In the present study, we identified the frequencies of genetic alterations of STAT3/CDK2/4/6 in multiple cancer types, identified the gene signature as oncogenic prognosticators of CAFs and tumor immune infiltration, and poor prognoses of clinical cancer cohorts. Our results demonstrated that the expression of CDK2/4/6 was altered in various cancers and is associated with both shorter OS and DFS of the cancer patients. We found CDK2/4/6 expression was particularly up-regulated in melanoma, glioblastoma, breast, colon, lung adenocarcinoma, head and neck, pancreatic, liver, and prostate cancer cohorts, compared to the adjacent normal tissues. Aberrant CDK2/4/6 expression may enhance cancer progression, in part, through influence on mechanisms that maintain cell cycle progression. Furthermore, it is worth noting that genetic alterations in CDK2/4/6 are associated with a poorer prognosis of the cancer cohorts. Indeed, we found that genetic alterations in CDK2/4/6 co-occurred with a number of other genetic alterations in the cancer cohorts. While we have yet to establish a cause of the co-occurrence relationship here, the genetic alterations in CDK2/4/6 could conceivably synergize with the observed gene alteration co-occurrence to promote tumor progression and hence could be responsible for the observed poorer survival of the CDK2/4/6 altered cohorts than the non-altered cohorts. This result is consistent with our previous study which suggested that targeting STAT3 and CDK2/4/6 is an attractive strategy for arresting cell growth in multiple cancers [52].

Evaluation of PPI networks is very useful for predicting biological processes associated with gene signatures and disease development [53]. The PPI network analysis in this study, suggest CDK2/4/6/STAT3 may directly interact with factors that promote tumorigenesis and immune response, such as AKT, EGFR, IL-6, IL-10, JAK1/2/3, CKS1B, CDT1, RB1, PLK1, ESP ORC2/3/4/5/6 (Supplementary Tables S1 and S2). The functions of these genes are found to be primarily related to cell cycle progressions, cancer development, and inflammatory and immune response [54]. The previous study has implicated the origin recognition complex (ORC) in the development of multiple cancers [55]. In addition, our KEGG pathways and gene ontologies studies showed that the CDK2/4/6 and STAT3 clustering PPI network were associated with pathways and processes involved in the cell cycle, DNA replication, cell communications, immune response, and cancers. Our findings are supported by a preclinical study that reported that increased expression of STAT3 led to concurrent increases in expressions of cytokines and growth factors (IL-6, IL-10, transforming growth factor (TGF)-β, and VEGF) [56]. Taking together, these results not only pointed out the potential roles of aberrant CDK2/4/6 expression in the initiation and development of multiple cancer but also suggest that STAT3 and CDK2/4/6 expression may alter tumor immune microenvironment and hence involved in cancer immune responses.

Tumor immune/inflammatory cell infiltrations are indicators of host immune responses to cancer cells [57,58]. We reasoned that since CDK2/4/6 and STAT3 clustering networks were enriched in cancer and inflammatory/immune-related pathways, then their hyper-expression levels in multiple cancers may be correlated with tumor immune infiltration. To this end, we investigated associations of STAT3/CDK2/4/6 expressions with tumor-immune infiltration across the TCGA dataset. We found that infiltration of macrophages, dendritic cells, CD4+ T cells, CD8+ T cells, neutrophils, and B cells were positively correlated with STAT3/CDK2/4/6 expressions in lung adenocarcinoma, liver hepatocellular carcinoma, head and neck cancer, and breast invasive carcinoma cohorts, suggesting that CDK2/4/6/STAT3 may also reflect the immune status besides the disease prognosis. This observation is in concordance with our observations in the pathway enrichment analysis of the PPI clustering network. These findings, therefore, suggest that STAT3/CDK2/4/6 participates in the immune invasion of the above-mentioned cancers, thus providing a new window for monitoring the tumor immune microenvironment and may serve as a potential prognostic biomarker of an immune response in those cancer [57]. Our result is supported by preclinical studies which revealed that aberrant STAT3 expression mediates immunosuppression of tumor cells [59,60]. Findings from the present study may therefore be clinically useful in prognosis assessment and follow-up management of immunotherapy. In addition, targeting the STAT3/CDK2/4/6 signaling axis may provide a dual role of oncogene suppression and immunotherapeutic responses in multiple cancers.

Having established the negative correlation of tumor immune infiltration with CDK2/4/6 expression in GBM and melanoma, we wonder if there will be an association between these genes alterations and immune infiltration, to this ends we queried the correlation between different somatic copy number alterations of CDK2/4/6/STAT3 and immune cell infiltration in glioblastoma and melanoma. The results revealed a similar trend of the negative association of various immune cell infiltrations with CDK2, CDK4, and STAT3 SCNA in GBM and CDK2/4/6/STAT3 SCNA in melanoma. Collectively this study suggested that the immune cell infiltrations of GBM and melanoma are inversely associated with CDK2/4/6 and STAT3 expression or genetic alterations.

Two distinct mechanisms of tumor immune evasion have been revealed, indicating that some tumors have a high level of infiltration by cytotoxic T cells, but these T cells tend to be in a dysfunctional state and could not control tumor growth, while in other tumors immunosuppressive factors may prevent T cells from infiltrating tumors [9,61,62]. DNA methylation is a key epigenetic modification in the mammalian genomes which plays an important role in the regulation of gene expression and therefore can serve as a non-invasive biomarker for cancer diagnosis and prognosis [63]. Consequently, we found that differential-methylation and copy number alterations of STAT3/CDK2/4/6 are associated with dysfunctional T-cell phenotypes, high death risk, and short survival duration of multiple cancer cohorts, hence providing preliminary evidence for the use of STAT3/CDK2/4/6 signature for DNA methylation-based biomarkers of dysfunctional T-cell phenotypes.

Because of that, the tumor immune infiltration of T cell is closely associated with the efficiency of the immune checkpoint inhibitor therapy [64]; thus, we evaluated the impact of the expression of STAT3/CDK2/4/6 on the therapeutic outcome of immune checkpoint blockade. We found that patients with high expression of STAT3, CDK2**,** CDK4, or CDK6 yield poor clinical benefit to anti-PD1 or anti-CTLA4 therapy and had shorter survival time than those patients with low expression profiles. Glioblastoma is one of the most aggressive cancers, although data are not yet mature, preliminary studies do not show a clear-cut benefit of immunotherapy in glioblastoma [65], while clinical studies have demonstrated that CDK4/6 inhibitor alone was not an effective treatment for recurrent glioblastoma [66,67]. Thus, our study showed that the STAT3/CDK2/4/6 signature not only regulates immune cell infiltration but also affects the benefit for cancer patients of immune checkpoint blockade and provides a rationale for the combination of STAT3/CDK2/4/6 antagonist and immunological checkpoint inhibitors. In line with this rationale, a combined preclinical and clinical study [68] have reported that PD-L1 protein abundance and tumor-infiltrating lymphocyte is regulated by cell cycle kinases and that the Inhibition of CDK4/6 increases PD-L1 protein and reduced the numbers of tumor-infiltrating lymphocytes in mouse and in human cancer specimens. Intriguingly, they found that a combination of CDK4/6 inhibitor with anti-PD-1 immunotherapy enhances tumor regression and markedly improves overall survival rates in mouse tumor models [68]. In addition, there are several ongoing trials testing combinations of CDK4/6 inhibitors with immunotherapy, including avelumab and pembrolizumab (e.g., NCT02778685; NCT02779751; and NCT03147287) [69].

The TME is a complex and heterogeneous ecosystem consisting of signaling molecules, tumor-infiltrating immune cells, extracellular matrix (ECM), CAFs, and tumor cells [70,71,72]. Genes that are highly expressed in the microenvironment are negatively associated with tumor purity [73]; therefore, our observation that STAT3 expression was negatively correlated with tumor purity in all tumor samples analyzed (Table 2) suggests its high expression in TMEs and justifies its positive correlation with tumor immune infiltration. In addition, we also found significant associations between STAT3/CDK2/4/6 expression profile and CAF infiltrations in multiple cancers (Figure 6A), and these positive correlations between CAF and STAT3/CDK2/4/6 expressions were also found to be associated with low cumulative survival of the cohorts (Figure 6B). CAFs are apoptotic-resistant and known to inhibit T-cell expansion, by secreting factors that upregulate the expression of checkpoint molecules in the TME, thus, hampering an effective anti-tumor response [74]. They promote tumorigenic and metastatic properties by secreting cytokines and remodeling the ECM [75,76]. Collectively, our study suggested that STAT3/CDK2/4/6 are important onco-immune signatures that play central roles in tumor progression, tumor immune invasion, poor prognoses, and it’s associated with poor response to immunotherapy; thus, concurrent targeting of this onco-immune signature together with immunotherapy may open up new windows for long-lasting, multilayer tumor control. This study may also provide background immunization details to assist in the design and follow-up of immunotherapies.

## 5. Conclusions

In conclusion, this study identified STAT3/CDK2/4/6 as an oncogenic prognosticator of cancer-associated fibroblasts and tumor immune infiltrations, and poor prognosis of multiple cancer cohorts. In addition, it’s associated with poor response to immunotherapy Genetic alteration of STAT3/CDK2/4/6 co-occurred with other gene alteration and are associated with poorer prognosis of the cohorts. This finding may therefore be clinically useful in designing appropriate therapeutic strategies, prognosis assessment, and follow-up management of immunotherapy in multiple cancer excepting melanoma and glioblastoma.

## Figures and Tables

**Table 1:** STAT3/CDK2/CDK4/CDK6 immunohistochemistry profile of cancer cohorts from human protein atlas database (HPA).

Cancer Types	Sample in HPA		Patient Age	Patient Gender		Tumor-Histology
	Total Sample	High Antibody Detected	Mean Age	Male n (%)	Female n (%)	Patient Tumor-Histology (%)
		**CDK2 (Antibody: CAB013115)**				
Breast	11	6 (54.54%)	54.16	-	6 (100%)	DCN (33.33%) and LCN (66.66%)
Head and Neck	4	4 (100 %)	56.75	2 (50.00%)	2 (50.00%)	HN-SCC (50.00%) and HN-ADC (50.00%)
Glioma	11	7 (63.63%)	58.14	5 (83.33%)	3 (16.66%)	HGG (57.14%), LGG (42.85%)
Colorectal	10	10 (100%)	69.90	6 (60.00%)	4 (40.00%)	C-ADC (70.00%), R-ADC (20.00%)
Prostate	10	5 (50.00%)	59.44	5 (100%)	-	HG_PA (60.00%) and LG_PA (40.00%)
Lung	10	6 (60.00%)	58.83	3 (50.00%)	3 (50.00%)	L-SSC (66.66%) and L-AND (33.33%)
Liver	11	4 (36.36%)	63.50	-	4 (100.00%)	CCN (25.00%) and HCN (75.00%)
Pancreatic	12	6 (50.00%)	64.16	3 (50.00%)	3 (50.00%)	PAC (100.00%)
		**CDK4 (Antibody: CAB013116)**				
Breast	11	9 (81.81%)	64.00	-	9 (100%)	DCN (55.55%) and LCN (44.44%)
Head and Neck	4	4 (100%)	71.5	3 (75.0%)	1 (25.00%)	HN-SCC (75.00%) and HN-ADC (35.00%)
Glioma	11	9 (81.81%)	48.11	5 55.55%)	4 (44.44%)	HGG (55.55%) and LGG (44.44%)
Colorectal	12	12 (100%)	79.50	6 (50.00%)	6 (50.00%)	C-ADC (66.66%) and R-ADC (33.33%)
Prostate	11	10 (90.90%)	58.80	10 (100%)	-	HG_PA (70.00%) and LG_PA (30.00%)
Lung	12	12 (100%)	67.58	7 (58.33%)	5 (41.66%)	L-SSC (58.33%) and L-AND (41.66%)
Liver	12	8 (66.66%)	63.25	5 62.55%)	3 (37.5%)	CCN (25.00%) and 6 HCN (75.00%)
Pancreatic	11	7 (63.63%)	63.71	4 (57.15%)	3 (42.85%)	PAC (100.00%)
		**CDK6 (Antibody: HPA002637)**				
Breast	12	3 (25.00%)	54.00	-	3 (100%)	DCN (75.00%) and LCN (25.00%)
Head and Neck	4	4 (100.00%)	58.25	1 (25%)	3 (75%)	HN-SCC (50.00%) and HN-ADC (50.00%)
Glioma	12	11 (91.66%)	44.58	3 (100%)	-	HGG (63.63%) and LGG (36.36%)
Colorectal	10	9 (90.00%)	61.60	6 (66.6%)	3 (33.33%)	C-ADC (66.66%) and R-ADC (6.33%)
Prostate	10	2 (20.00%)	66.00	2 (100%)	-	HG_PA (50.00%) and LG_PA (50.00%)
Lung	11	4 (36.36%)	61.00	2 (50%)	2 (50%)	L-SSC (75.00%) and L-AND (25.00%)
Liver	12	9 (75.00%)	62.77	5 (55.55%)	4 (44.5%)	CCN (66.66%) and HCN (33.33%)
Pancreatic	11	7 (63.63%)	62.00	3 (42.85%)	4 (57.1%)	PAC (100.00%)
		**STAT3 (Antibody: HPA001671)**				
Breast	11	11 (100%)	63.18	-	11 (100%)	DCN (72.72%) and LCN (27.27%)
Head and Neck	4	4 (100%)	70.50	3 (75.00%)	1 (25.00%)	HN-SCC (75.00%) and HN-ADC (25.00%)
Glioma	12	5 (41.66%)	45.60	2 (40.00%)	3 (60.00%)	HGG (80.00%) and LGG (20.00%)
Colorectal	12	12 (100%)	64.83	4 (33.33%)	8 (66.66%)	C-ADC (75.00%) and R-ADC (25.00%)
Prostate	10	9 (90.00%)	67.44	10 (100%)	-	HG_PA (88.88%) and LG_PA (11.11%)
Lung	12	6 (50.00%)	69.50	4 (66.66%)	2 (33.33%)	L-SSC (50.00%) and L-AND (50.00%)
Liver	11	4 (36.36%)	57.75	2 (50.00%)	2 (50.00%)	CCN (5000%) and 6 HCN (50.00%)
Pancreatic	9	6 (66.66%)	63.71	3 (50.00%)	3 (50.00%)	PAC (100.00%)

Key: DCN: duct carcinoma; LCN: lobular carcinoma; HN-SCC: head and neck squamous cell carcinoma; HN-ADC: head and neck adenocarcinoma; HGG: high grade glioma; LGG: low grade glioma; C-ADC: colon adenocarcinoma; R-ADC: rectum adenocarcinoma; HG_PA: high grade prostate adenocarcinoma; LG_PA: low grade prostate adenocarcinoma; L-SSC: lung squamous cell carcinoma; L-AND: lung adeno carcinoma; CCN: cholangiocarcinoma; HCN: hepatocellular carcinoma; PAC: pancreatic adenocarcinoma.

**Table 2:** Purity-corrected partial Spearman’s rho value and statistical significance of the correlations of STAT3/CDK2/CDK4/CDK6 expressions with immune infiltration level in diverse cancer types.

Cancer Types	Variable	CDK2		CDK4		CDK6		STAT3	
		rho-Value	*p*-Value	rho-Value	*p*-Value	rho-Value	*p*-Value	rho-Value	*p*-Value
**BRCA**	Purity	0.173772	3.46 × 10^−8^	0.093476	0.003164	−0.31833	7.21 × 10^−25^	−0.10635	0.000779
	B Cell	0.122448	0.000125	0.08921	0.005264	0.240495	2.54 × 10^−14^	0.066812	0.036797
	CD8+ T Cell	0.192216	1.41 × 10^−9^	0.032843	0.305355	0.391337	4.53 × 10^−37^	0.239179	3.65 × 10^−14^
	CD4+ T Cell	0.140028	1.31 × 10^−5^	0.042232	0.190619	0.320767	1.85 × 10^−24^	0.233229	2.38 × 10^−13^
	Macrophage	0.079207	0.012988	−0.01619	0.612245	0.257793	2.19 × 10^−16^	0.25456	5.28 × 10^−16^
	Neutrophil	0.233463	3.07 × 10^−13^	0.096267	0.002962	0.391841	2.93 × 10^−36^	0.313132	4.44 × 10^−23^
	Dendritic Cell	0.169013	1.58 × 10^−7^	0.113947	0.00043	0.384255	8.01 × 10^−35^	0.202448	2.97 × 10^−10^
**GBM**	Purity	0.286993	2.19 × 10^−9^	0.430653	2.39 × 10^−20^	0.192757	7.15 × 10^−5^	−0.16588	0.000652
	B Cell	−0.05908	0.228103	0.01657	0.73552	0.02255	0.645725	−0.00829	0.865847
	CD8+ T Cell	−0.03756	0.443695	−0.04642	0.343759	0.138459	0.004568	−0.14558	0.002851
	CD4+ T Cell	−0.05077	0.300396	−0.02841	0.562404	−0.0257	0.600353	0.277782	7.64 × 10^−9^
	Macrophage	−0.01146	0.815223	−0.02026	0.679627	−0.03727	0.447329	0.056771	0.246809
	Neutrophil	0.089753	0.006772	0.052908	0.28049	−0.16547	0.000683	0.175587	0.00031
	Dendritic Cell	0.200704	3.58 × 10^−5^	0.023584	0.630667	−0.12206	0.012513	0.43801	5.06 × 10^−21^
**HNSC**	Purity	0.229737	2.51 × 10^−7^	0.304761	4.69 × 10^−12^	−0.06379	0.157308	−0.01693	0.707708
	B Cell	0.110468	0.015902	0.124189	0.00667	−0.21932	1.36 × 10^−6^	0.230367	3.75 × 10^−7^
	CD8+ T Cell	0.092891	0.043236	0.07504	0.102739	−0.25071	3.16 × 10^−8^	0.235486	2.14 × 10^−7^
	CD4+ T Cell	0.299532	2.09 × 10^−11^	0.140472	0.002036	0.189753	2.86 × 10^−5^	0.456441	4.47 × 10^−26^
	Macrophage	0.146554	0.001238	0.190636	2.47 × 10^−5^	−0.013	0.775631	0.21852	1.24 × 10^−6^
	Neutrophil	0.215968	1.84 × 10^−6^	0.021853	0.6333	0.082199	0.072281	0.346725	5.62 × 10^−15^
	Dendritic Cell	0.253224	1.73 × 10^−8^	0.103755	0.00272	0.040238	0.378066	0.387553	1.01 × 10^−18^
**LIHC**	Purity	0.181946	0.000672	0.069596	0.196552	−0.11342	0.034946	−0.23257	1.24 × 10^−5^
	B Cell	0.397861	1.70 × 10^−14^	0.446746	2.80 × 10^−18^	0.077473	0.151618	0.167119	0.001869
	CD8+ T Cell	0.300309	1.47 × 10^−8^	0.327963	5.11 × 10^−10^	0.024258	0.654848	0.128993	0.016998
	CD4+ T Cell	0.423424	2.13 × 10^−16^	0.379031	3.39 × 10^−13^	0.062486	0.247738	0.348425	2.97 × 10^−11^
	Macrophage	0.476735	9.42 × 10^−21^	0.51059	4.90 × 10^−24^	0.097956	0.070829	0.359076	8.16 × 10^−12^
	Neutrophil	0.477554	4.69 × 10^−21^	0.368888	1.46 × 10^−12^	0.076032	0.158794	0.448825	1.67 × 10^−18^
	Dendritic Cell	0.480477	4.86 × 10^−21^	0.482455	3.18 × 10^−21^	0.052521	0.334277	0.285271	8.68 × 10^−08^
**LUAD**	Purity	0.06579	0.144252	0.060096	0.182364	−0.16182	0.000304	0.007492	0.868083
	B Cell	−0.04115	0.366308	−0.10408	0.022022	−0.03669	0.420564	0.119856	0.008302
	CD8+ T Cell	0.146119	0.001222	0.006762	0.881683	0.275158	6.57 × 10^−10^	0.128065	0.004647
	CD4+ T Cell	0.071922	0.001145	−0.09706	0.032783	0.146028	0.001275	0.167818	0.000208
	Macrophage	0.03284	0.470574	−0.01899	0.67652	0.207622	4.01 × 10^−6^	0.158864	0.000445
	Neutrophil	0.27285	1.08 × 10^−9^	0.078989	0.082887	0.345911	5.06 × 10^−15^	0.219087	1.16 × 10^−6^
	Dendritic Cell	0.134315	0.002949	0.021403	0.637169	0.208912	3.25 × 10^−6^	0.179555	6.64 × 10^−5^
**SKCM**	Purity	0.134716	0.003873	0.33632	1.42 × 10^−13^	0.208989	6.48 × 10^−6^	−0.09559	0.040865
	B Cell	−0.04378	0.355269	0.035404	0.454766	0.092088	0.051436	0.190704	4.85 × 10^−5^
	CD8+ T Cell	−0.025	0.601752	−0.09342	0.050716	0.273715	5.76 × 10^−9^	0.325874	2.70 × 10^−12^
	CD4+ T Cell	−0.14917	0.001582	−0.05216	0.271664	0.151926	0.00129	0.276917	2.71 × 10^−9^
	Macrophage	−0.28478	6.72 × 10^−10^	−0.06167	0.190155	0.252014	5.42 × 10^−8^	0.32638	1.05 × 10^−12^
	Neutrophil	−0.18426	8.13 × 10^−5^	−0.0556	0.238103	0.455928	1.39 × 10^−24^	0.500159	5.51 × 10^−30^
	Dendritic Cell	−0.08306	0.079749	−0.00475	0.920327	0.188403	6.24 × 10^−5^	0.373995	2.97 × 10^−16^

Key: rho-value = purity-corrected partial Spearman’s rho value; *p*-value = statistical significance. SKCE: Skin cutaneous melanoma; LUAD: Lung adenocarcinoma; LIHC: Liver hepatocellular carcinoma; HNSC: Head and neck cancer; GBM: glioblastoma; BRCA: Breast invasive carcinoma.

**Table 3:** Co-occurrence of gene alteration in cancer cohorts with STAT3/CDK2/2/4/6 genetic alteration.

S/N	Genes ID	Cytoband	Altered Group	Unaltered Group	Log Ratio	*p*-Value	*q*-Value	Enriched in
	**Cyclin Dependent Kinase 2**							
1	FGD5	3p25.1	19 (15.32%)	226 (2.19%)	2.81	6.99 × 10^−11^	7.60 × 10^−7^	Altered group
2	MMS22L	6q16.1	17 (13.71%)	178 (1.73%)	2.99	1.23 × 10^−10^	7.60 × 10^−7^	Altered group
3	LRP5	11q13.2	19 (15.32%)	237 (2.30%)	2.74	1.49 × 10^−10^	7.60 × 10^−7^	Altered group
4	PALM2-AKAP2	9q31.3	17 (13.71%)	181 (1.76%)	2.97	1.56 × 10^−10^	7.60 × 10^−7^	Altered group
5	GTF3C2	2p23.3	15 (12.10%)	135 (1.31%)	3.21	2.37 × 10^−10^	7.67 × 10^−7^	Altered group
6	PAX8	2q14.1	12 (9.68%)	72 (0.70%)	3.79	2.53 × 10^−10^	7.67 × 10^−7^	Altered group
7	MFHAS1	8p23.1	13 (10.48%)	93 (0.90%)	3.54	3.11 × 10^−10^	7.67 × 10^−7^	Altered group
8	SENP5	3q29	13 (10.48%)	94 (0.91%)	3.52	3.51 × 10^−10^	7.67 × 10^−7^	Altered group
9	PRDM9	5p14.2	25 (20.16%)	461 (4.47%)	2.17	3.86 × 10^−10^	7.67 × 10^−7^	Altered group
10	LRRFIP2	3p22.2	13 (10.48%)	95 (0.92%)	3.51	3.95 × 10^−10^	7.67 × 10^−7^	Altered group
	**Cyclin Dependent Kinase 4**							
1	EGFR	7p11.2	39 (13.68%)	357 (3.52%)	1.96	2.25 × 10^−12^	4.38 × 10^−8^	Altered group
2	ZNF19	16q22.2	18 (6.32%)	73 (0.72%)	3.13	3.28 × 10^−11^	3.19 × 10^−7^	Altered group
3	NUP107	12q15	19 (6.67%)	128 (1.26%)	2.4	1.69 × 10^−8^	5.45 × 10^−5^	Altered group
4	KLHL9	9p21.3	15 (5.26%)	75 (0.74%)	2.83	1.76× 10^−8^	5.45 × 10^−5^	Altered group
5	ATP13A5	3q29	25 (8.77%)	227 (2.24%)	1.97	2.23× 10^−8^	5.45 × 10^−5^	Altered group
6	TENM2	5q34	35 (12.28%)	418 (4.12%)	1.58	2.27× 10^−8^	5.45 × 10^−5^	Altered group
7	MYPN	10q21.3	26 (9.12%)	247 (2.43%)	1.91	2.68× 10^−8^	5.45 × 10^−5^	Altered group
8	B4GALNT1	12q13.3	15 (5.26%)	78 (0.77%)	2.78	2.79× 10^−08^	5.45 × 10^−05^	Altered group
9	PTPRH	19q13.42	24 (8.42%)	214 (2.11%)	2	3.15× 10^−08^	5.45 × 10^−05^	Altered group
10	NEMF	14q21.3	18 (6.32%)	120 (1.18%)	2.42	3.53× 10^−08^	5.45 × 10^−05^	Altered group
	**Cyclin Dependent Kinase 6**							
1	TP53	17p13.1	159 (62.6%)	3680 (36.14%)	0.79	2.75 × 10^−17^	5.35 × 10^−13^	Altered group
2	CFAP47	Xp21.1	43 (16.93%)	399 (3.92%)	2.11	3.41 × 10^−15^	3.31 × 10^−11^	Altered group
3	CUBN	10p13	51 (20.08%)	625 (6.14%)	1.71	2.03 × 10^−13^	1.31 × 10^−9^	Altered group
4	KBTBD7	13q14.11	20 (7.87%)	81 (0.80%)	3.31	2.90 × 10^−13^	1.41 × 10^−9^	Altered group
5	EYS	6q12	39 (15.35%)	385 (3.78%)	2.02	4.73 × 10^−13^	1.84 × 10^−9^	Altered group
6	FAT3	11q14.3	59 (23.23%)	839 (8.24%)	1.5	7.48 × 10^−13^	2.23 × 10^−9^	Altered group
7	SPTBN4	19q13.2	34 (13.39%)	298 (2.93%)	2.19	8.39 × 10^−13^	2.23 × 10^−9^	Altered group
8	TCERG1L	10q26.3	21 (8.27%)	99 (0.97%)	3.09	9.29 × 10^−13^	2.23 × 10^−9^	Altered group
9	ATP2B1	12q21.33	25 (9.84%)	153 (1.50%)	2.71	1.03 × 10^−12^	2.23 × 10^−9^	Altered group
10	UBA6	4q13.2	24 (9.45%)	141 (1.38%)	2.77	1.38 × 10^−12^	2.68 × 10^−9^	Altered group
	**Signal Transducer and Activator of Transcription 3**							
1	NEURL4	17p13.1	39 (17.89%)	177 (1.73%)	3.37	5.90 × 10^−26^	1.15 × 10^−21^	Altered group
2	ARHGAP5	14q12	38 (17.43%)	202 (1.98%)	3.14	3.78 × 10^−23^	3.67 × 10^−19^	Altered group
3	DSG1	18q12.1	38 (17.43%)	208 (2.04%)	3.1	9.42 × 10^−23^	6.10 × 10^−19^	Altered group
4	PCDHGB6	5q31.3	34 (15.60%)	157 (1.54%)	3.34	1.81 × 10^−22^	8.80 × 10^−19^	Altered group
5	CEP350	1q25.2	44 (20.18%)	318 (3.11%)	2.7	5.68 × 10^−22^	2.21 × 10^−18^	Altered group
6	MED13	17q23.2	40 (18.35%)	255 (2.50%)	2.88	9.18 × 10^−22^	2.97 × 10^−18^	Altered group
7	HMCN1	1q25.3	67 (30.73%)	846 (8.28%)	1.89	5.92 × 10^−21^	1.64 × 10^−17^	Altered group
8	DOCK8	9p24.3	39 (17.89%)	255 (2.50%)	2.84	7.08 × 10^−21^	1.72 × 10^−17^	Altered group
9	DNMBP	10q24.2	34 (15.60%)	180 (1.76%)	3.15	8.24 × 10^−21^	1.78 × 10^−17^	Altered group
10	MAP1B	5q13.2	40 (18.35%)	277 (2.71%)	2.76	1.37 × 10^−20^	2.65 × 10^−17^	Altered group

## Data Availability

The datasets generated and/or analyzed in this study are available on reasonable request.

## Supplementary Materials

The following are available online at https://www.mdpi.com/2072-6694/13/5/954/s1, Table S1: Proteins directly interacting with CDK2/4/6. Table S2: Proteins directly interacting with STAT3. Figure S1: Scatterplots showing the correlation of STAT3 expression with immune infiltration level in diverse cancer types. SKCE; Skin cutaneous melanoma, LUAD; Lung adenocarcinoma; LIHC; Liver hepatocellular carcinoma; HNSC; Head and neck cancer, GBM; glioblastoma, BRCA; Breast invasive carcinoma. Figure S2: Scatterplots showing the correlation of CDK2 expression with immune infiltration level in diverse cancer types. SKCE; Skin cutaneous melanoma, LUAD; Lung adenocarcinoma; LIHC; Liver hepatocellular carcinoma; HNSC; Head and neck cancer, GBM; glioblastoma, BRCA; Breast invasive carcinoma. Figure S3: Scatterplots showing the correlation of CDK4 expression with immune infiltration level in diverse cancer types. SKCE; Skin cutaneous melanoma, LUAD; Lung adenocarcinoma; LIHC; Liver hepatocellular carcinoma; HNSC; Head and neck cancer, GBM; glioblastoma, BRCA; Breast invasive carcinoma. Figure S4: Scatterplots showing the correlation of CDK6 expression with immune infiltration level in diverse cancer types. SKCE; Skin cutaneous melanoma, LUAD; Lung adenocarcinoma; LIHC; Liver hepatocellular carcinoma; HNSC; Head and neck cancer, GBM; glioblastoma, BRCA; Breast invasive carcinoma. Figure S5: Box plots showing tumor immune infiltration levels in GBM patients with different somatic copy number alterations for CDK2/CDK4/CDK6/STAT3. The infiltration abundance in every SCNA category was compared to the diploid/normal. * *p* < 0.05; ** *p* < 0.01; *** *p* < 0.001. Figure S6: Graphical data representation of association between CNA of STAT3/CDK2/4/6 and dysfunctional T-cell phenotypes and prognostic relevance in multiple cancers.
