# Supplementary material for: Multi-Omics Data Analysis of Gene Expressions and Alterations, Cancer-Associated Fibroblast and Immune Infiltrations, Reveals the Onco-Immune Prognostic Relevance of STAT3/CDK2/4/6 in Human Malignancies
Source: Cancers (Basel). 2021 Feb 25;13(5):954. doi: 10.3390/cancers13050954 (PMC7956610; doi:10.3390/cancers13050954)
Supplement: Supplementary file 1 [file cancers-13-00954-s001.pdf]

cancers

MDPI

*Article*

# Supplemental Materials: Multi-Omics Data Analysis of Gene Expressions and Alterations, Cancer-Associated Fibroblast and Immune Infiltrations, Reveals the Onco-Immune Prognostic Relevance of STAT3/CDK2/4/6 in Human Malignancies

**Bashir Lawal, Li-Ching Lin, Jih-Chin Lee, Jia-Hong Chen, Tanios S. Bekaii-Saab, Alexander T.H. Wu and Ching-Liang Ho**

**Table S1.** Proteins directly interacting with CDK2/4/6.

| CDK2 | | CDK4 | | CDK6 | |
|---|---|---|---|---|---|
| Interactive Proteins | Interactive Scores | Interactive Proteins | Interactive Scores | Interactive Proteins | Interactive Scores |
| PLK1 | 0.603 | PLK1 | 0.507 | MCM6 | 0.453 |
| ESPL1 | 0.707 | MCM6 | 0.543 | PLK1 | 0.455 |
| CDKN2C | 0.744 | ORC2 | 0.551 | ORC2 | 0.524 |
| MAD2L1 | 0.775 | ESPL1 | 0.598 | MCM4 | 0.527 |
| CDK4 | 0.926 | MCM4 | 0.602 | CDT1 | 0.556 |
| ORC5 | 0.936 | MAD2L1 | 0.619 | MAD2L1 | 0.597 |
| ORC3 | 0.937 | MCM5 | 0.672 | MCM7 | 0.598 |
| ORC6 | 0.954 | MCM7 | 0.693 | ESPL1 | 0.609 |
| MCM6 | 0.969 | CDT1 | 0.746 | MCM5 | 0.612 |
| CDK6 | 0.974 | ORC3 | 0.884 | MCM2 | 0.928 |
| MCM7 | 0.979 | MCM2 | 0.884 | CKS1B | 0.963 |
| E2F1 | 0.985 | CKS1B | 0.972 | E2F1 | 0.973 |
| MCM5 | 0.988 | CDK6 | 0.975 | RB1 | 0.989 |
| ORC2 | 0.991 | E2F1 | 0.977 | CDKN1B | 0.995 |
| CDT1 | 0.993 | CDKN1B | 0.997 | CDKN2C | 0.999 |
| MCM2 | 0.994 | CDKN2C | 0.997 | | |
| MCM4 | 0.995 | RB1 | 0.999 | | |
| CDKN1B | 0.999 | | | | |
| CKS1B | 0.999 | | | | |
| RB1 | 0.999 | | | | |

**Table S2.** Proteins directly interacting with STAT3.

| Query Protein | Interactive Proteins | Interactive score |
|---|---|---|
| **STAT3** | AKT1 | 0.994 |
| **STAT3** | EGFR | 0.998 |
| **STAT3** | IL10 | 0.995 |
| **STAT3** | IL6 | 0.997 |
| **STAT3** | JAK1 | 0.997 |
| **STAT3** | JAK2 | 0.998 |
| **STAT3** | JAK3 | 0.994 |
| **STAT3** | SOCS3 | 0.995 |
| **STAT3** | SRC | 0.995 |
| **STAT3** | EGF | 0.984 |
| **STAT3** | GSK3B | 0.684 |
| **STAT3** | KDR | 0.789 |
| **STAT3** | MTOR | 0.989 |
| **STAT3** | NOS3 | 0.695 |
| **STAT3** | SRC | 0.995 |
| **STAT3** | STAT5B | 0.961 |
| **STAT3** | STAT5A | 0.976 |
| **STAT3** | VEGFA | 0.994 |

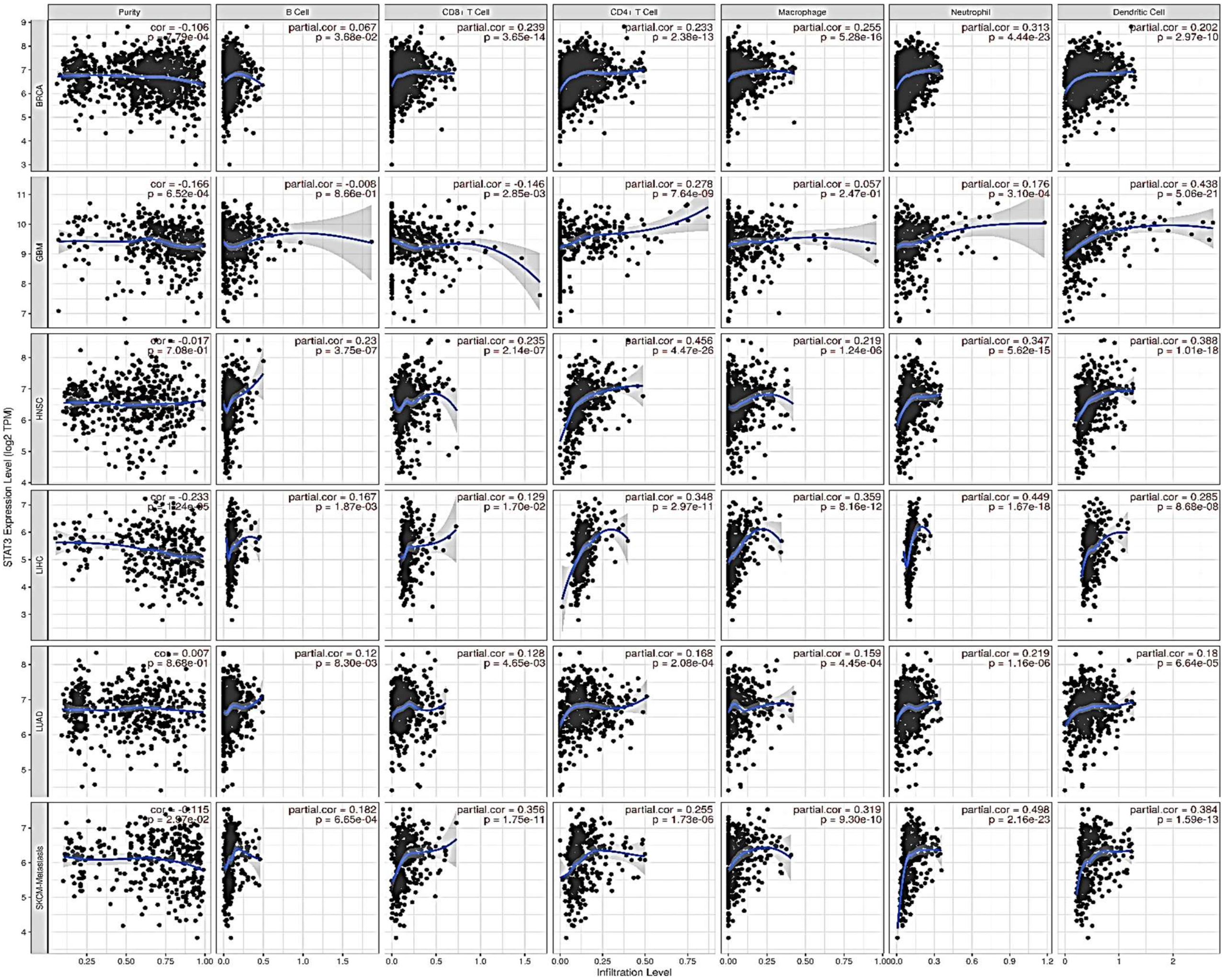


**Figure S1.** Scatterplots showing the correlation of STAT3 expression with immune infiltration level in diverse cancer types. SKCE; Skin cutaneous melanoma, LUAD; Lung adenocarcinoma; LIHC; Liver hepatocellular carcinoma; HNSC; Head and neck cancer, GBM; glioblastoma, BRCA; Breast invasive carcinoma.

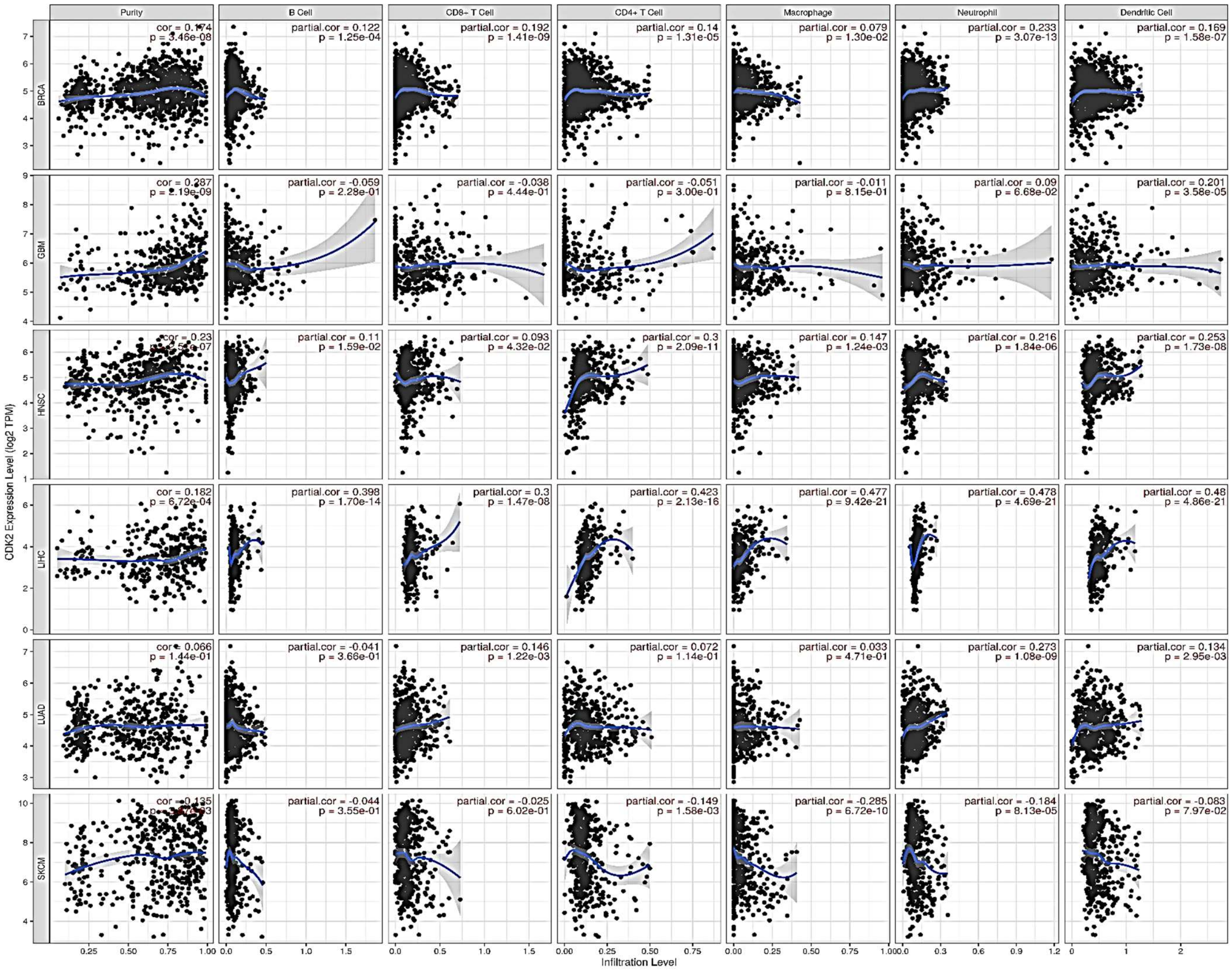


**Figure S2.** Scatterplots showing the correlation of CDK2 expression with immune infiltration level in diverse cancer types. SKCE; Skin cutaneous melanoma, LUAD; Lung adenocarcinoma; LIHC; Liver hepatocellular carcinoma; HNSC; Head and neck cancer, GBM; glioblastoma, BRCA; Breast invasive carcinoma.

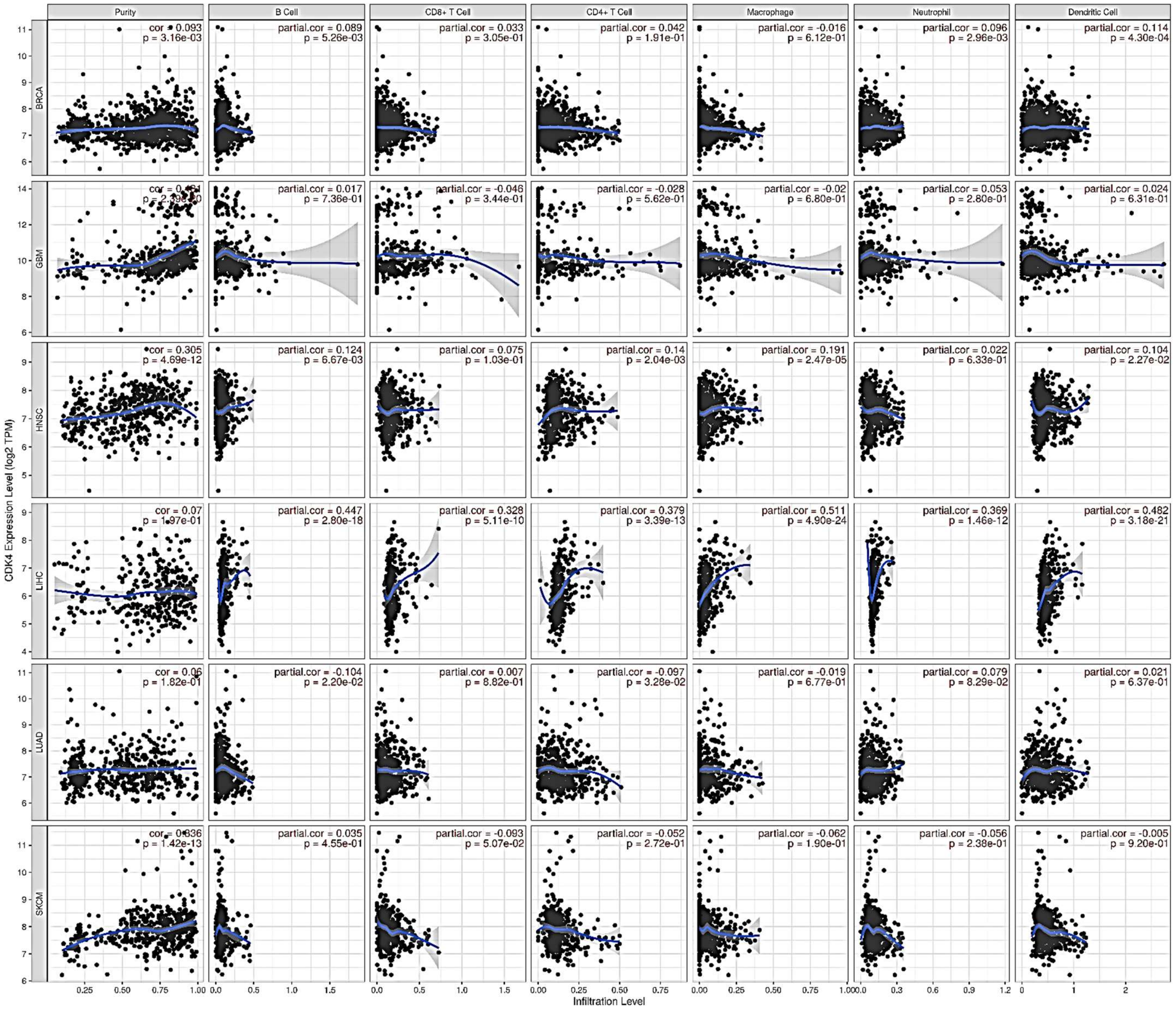


**Figure S3.** Scatterplots showing the correlation of CDK4 expression with immune infiltration level in diverse cancer types. SKCE; Skin cutaneous melanoma, LUAD; Lung adenocarcinoma; LIHC; Liver hepatocellular carcinoma; HNSC; Head and neck cancer, GBM; glioblastoma, BRCA; Breast invasive carcinoma.

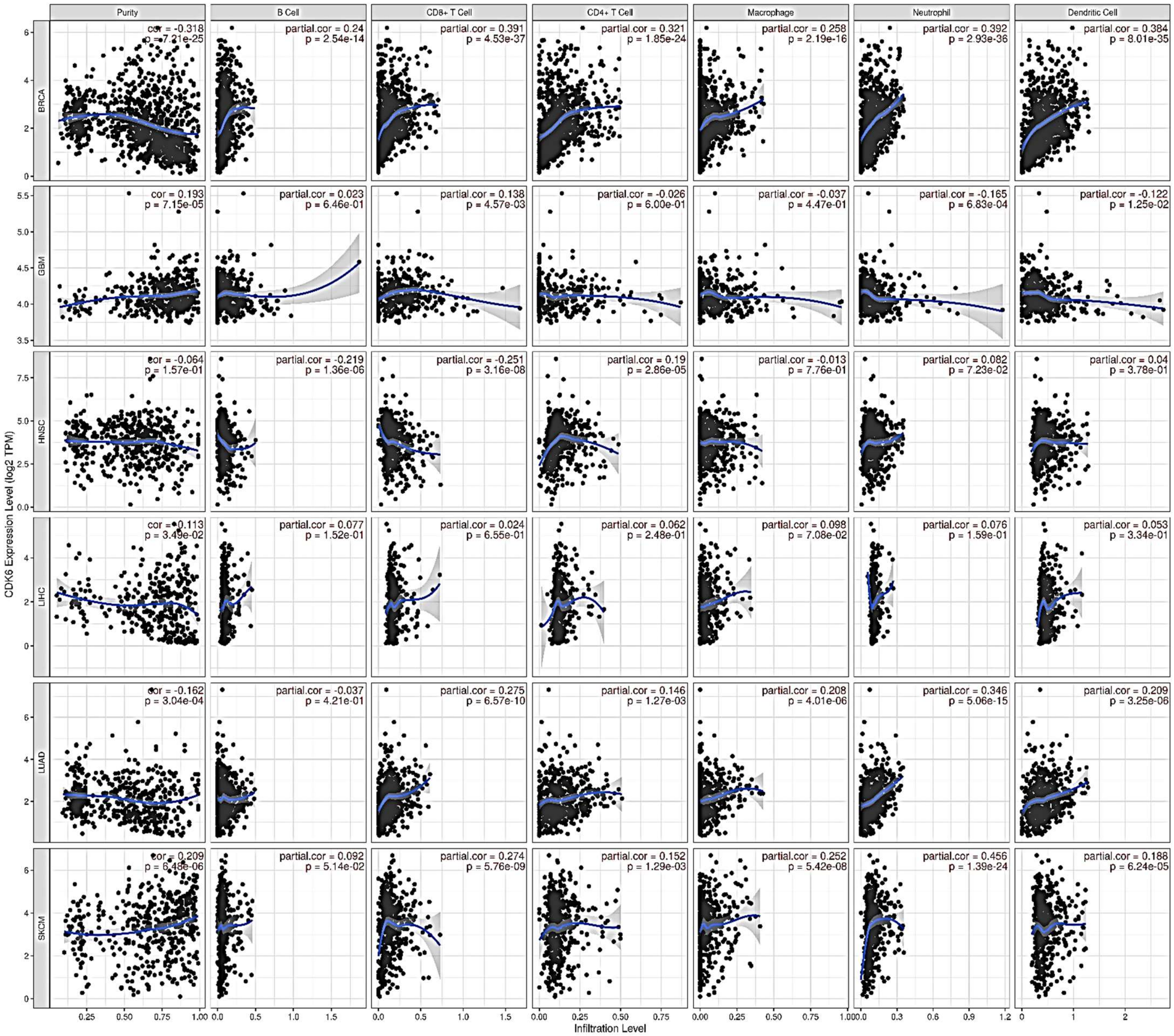


**Figure S4.** Scatterplots showing the correlation of CDK6 expression with immune infiltration level in diverse cancer types. SKCE; Skin cutaneous melanoma, LUAD; Lung adenocarcinoma; LIHC; Liver hepatocellular carcinoma; HNSC; Head and neck cancer, GBM; glioblastoma, BRCA; Breast invasive carcinoma.

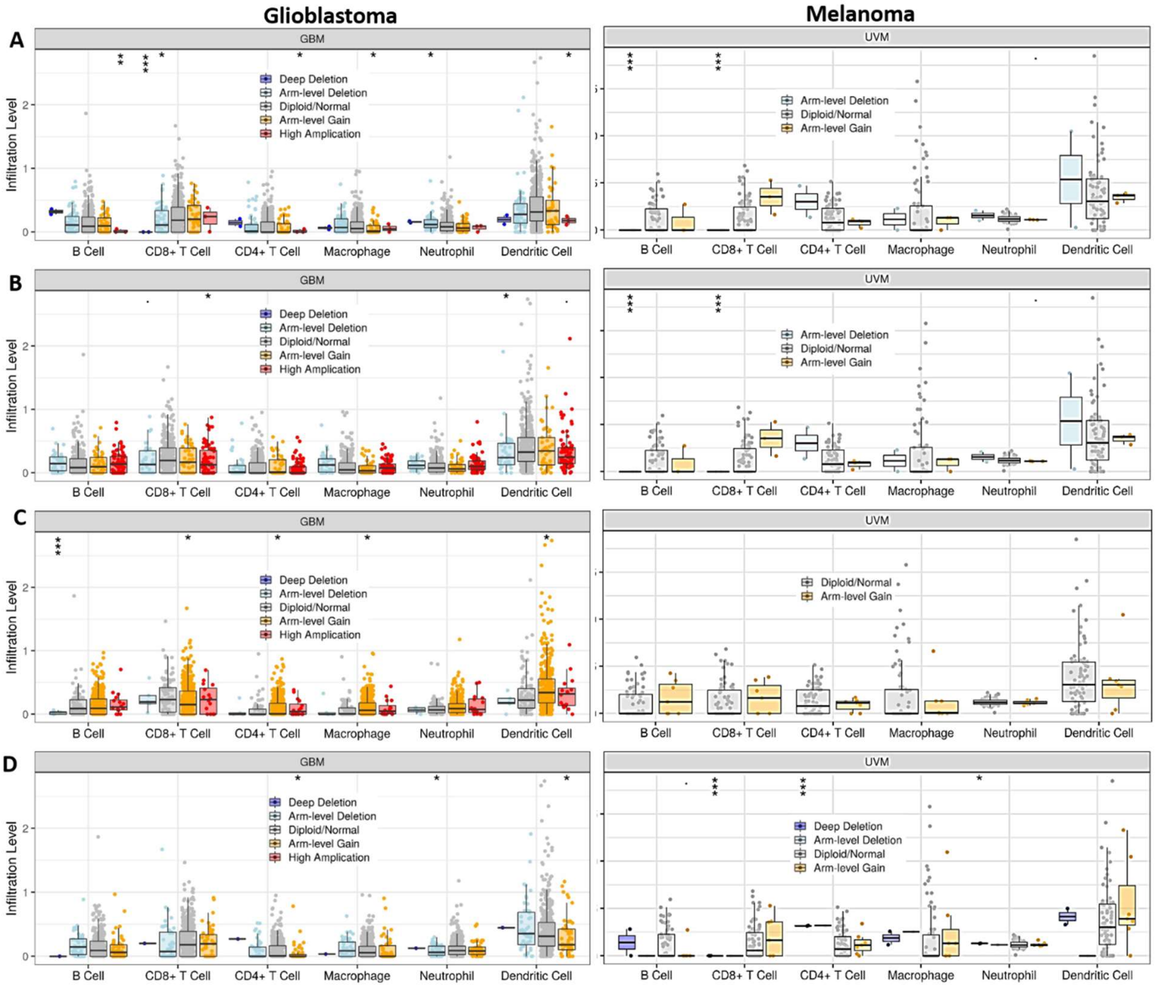


**Figure S5.** Box plots showing tumor immune infiltration levels in GBM patients with different somatic copy number alterations for CDK2/CDK4/CDK6/STAT3. The infiltration abundance in every SCNA category was compared to the diploid/normal. * $p < 0.05$; ** $p < 0.01$; *** $p < 0.001$.

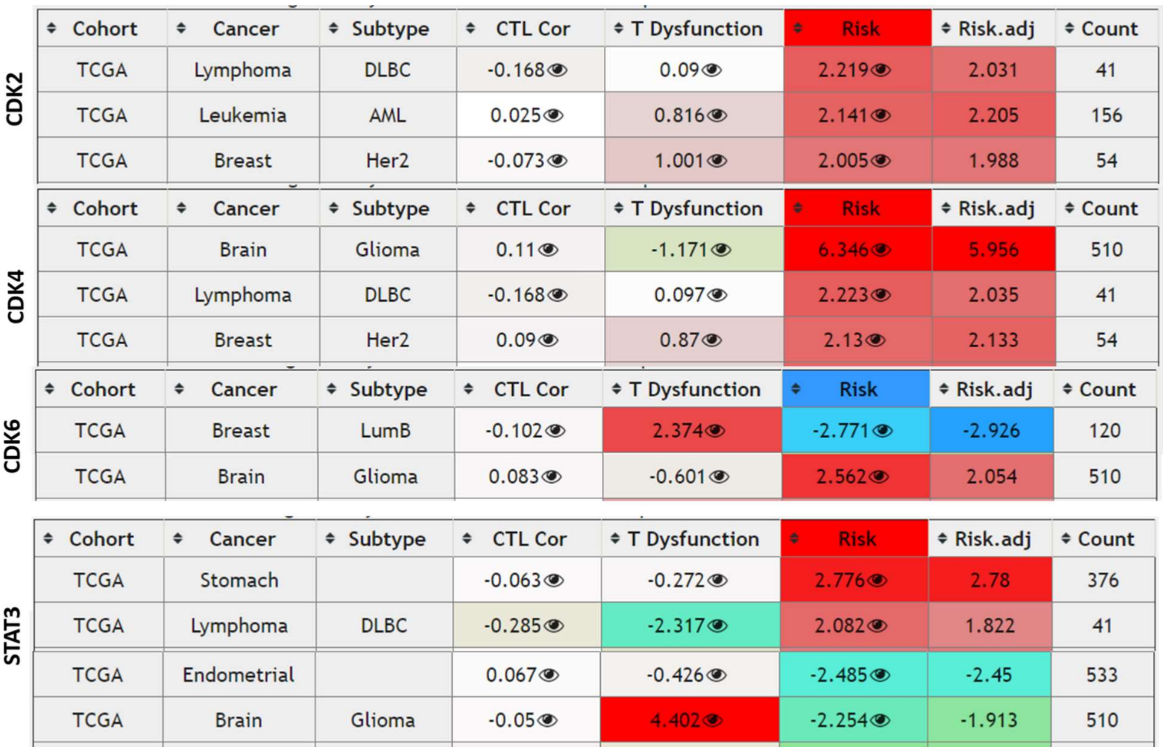

**CDK2**

| Cohort | Cancer | Subtype | CTL Cor | T Dysfunction | Risk | Risk.adj | Count |
|---|---|---|---|---|---|---|---|
| TCGA | Lymphoma | DLBC | -0.168 | 0.09 | 2.219 | 2.031 | 41 |
| TCGA | Leukemia | AML | 0.025 | 0.816 | 2.141 | 2.205 | 156 |
| TCGA | Breast | Her2 | -0.073 | 1.001 | 2.005 | 1.988 | 54 |

**CDK4**

| Cohort | Cancer | Subtype | CTL Cor | T Dysfunction | Risk | Risk.adj | Count |
|---|---|---|---|---|---|---|---|
| TCGA | Brain | Glioma | 0.11 | -1.171 | 6.346 | 5.956 | 510 |
| TCGA | Lymphoma | DLBC | -0.168 | 0.097 | 2.223 | 2.035 | 41 |
| TCGA | Breast | Her2 | 0.09 | 0.87 | 2.13 | 2.133 | 54 |

**CDK6**

| Cohort | Cancer | Subtype | CTL Cor | T Dysfunction | Risk | Risk.adj | Count |
|---|---|---|---|---|---|---|---|
| TCGA | Breast | LumB | -0.102 | 2.374 | -2.771 | -2.926 | 120 |
| TCGA | Brain | Glioma | 0.083 | -0.601 | 2.562 | 2.054 | 510 |

**STAT3**

| Cohort | Cancer | Subtype | CTL Cor | T Dysfunction | Risk | Risk.adj | Count |
|---|---|---|---|---|---|---|---|
| TCGA | Stomach | | -0.063 | -0.272 | 2.776 | 2.78 | 376 |
| TCGA | Lymphoma | DLBC | -0.285 | -2.317 | 2.082 | 1.822 | 41 |
| TCGA | Endometrial | | 0.067 | -0.426 | -2.485 | -2.45 | 533 |
| TCGA | Brain | Glioma | -0.05 | 4.402 | -2.254 | -1.913 | 510 |

**Figure S6.** Graphical data representation of association between CNA of STAT3/CDK2/4/6 and dysfunctional T-cell phenotypes and prognostic relevance in multiple cancers.
